# Inventions and patenting in Africa: Empirical trends from 1970 to 2010

**DOI:** 10.1111/jwip.12139

**Published:** 2019-11-25

**Authors:** Gregory D. Graff, Philip G. Pardey

**Affiliations:** ^1^ Department of Agricultural and Resource Economics Colorado State University Fort Collins Colorado; ^2^ Department of Technology Management and Economics Chalmers University of Technology Gothenburg Sweden; ^3^ International Science and Technology Practice and Policy (InSTePP) University of Minnesota St. Paul Minnesota; ^4^ Department of Applied Economics University of Minnesota St. Paul Minnesota

**Keywords:** Africa, intellectual property rights, international technology transfer, patent families, patent offices

## Abstract

Economic development is increasingly dependent upon on utilizing new knowledge to innovate and create value, even in traditional industries and in low‐income countries. This analysis uses evidence on patent families to assess innovation activity throughout sub‐Saharan Africa. We find patent activity in sub‐Saharan Africa—both by African inventors and by foreign inventors—is comparable to similar regions around the world, when conditioned on economic size. Patent filings in Africa have grown, particularly, since the mid‐1990s, but at different rates within different African jurisdictions. Types of technologies being patented in Africa have remained stable over 30 years, with most in pharmaceuticals, chemistry, biotechnology, and engineering. The majority of patent filings in Africa are from Europe, the United States, and other high income countries. Yet, in South Africa, between 15% and 20% of patent filings are by residents of South Africa, and 3% are from other developing and emerging economies. Only a small share of inventions globally are made in sub‐Saharan Africa, but for those inventions that do arise in Africa, foreign filings are made widely outside of Africa.

## 1 INTRODUCTION

The world's first patent statute—adopted in the city‐state of Venice in 1474—was an early preindustrial economic development policy. By contrast, the globalized patent system of today may seem far removed from the economic development goals of low‐ and middle‐income countries, such as those of sub‐Saharan Africa (hereafter, interchangeably as Africa). This is due, in large part, to the increasing asymmetry in technological capabilities that have grown, since the Industrial Revolution, between inventors in leading industrialized countries and technological followers in all other countries. Yet, economic development is increasingly a question of utilizing knowledge to innovate, solve problems, and create value, even in traditional industries and even in least‐developed countries. This is evidenced by the contributions of new knowledge—in the form of vaccines, improved crop varieties, or mobile telephone and data services—to the livelihoods of millions. Innovation has played an important role in meeting several of the Millennium Development Goals, and the need for further innovation is integral to the framing of the Sustainable Development Goals (United Nations, 2017).

Scholars and policymakers are divided on the role of patents in economic development policy (Barton et al., 2002; Maskus, 2000; Siebeck, Evenson, Lesser, & Primo Braga, 1990). The negotiation, adoption, and implementation of common minimum standards under the Trade Related Intellectual Property Rights (TRIPS) agreement as part of the World Trade Organization (WTO) Treaty provided ample context for this debate over the past three decades (Blakeney & Mengistie, 2011; Diwan & Rodrik, 1991), yet it is a debate that has run for far longer (May & Sell, 2006).

Some argue that intellectual property rights (IPRs) in developing countries are counterproductive to economic development. The first and strongest argument in this vein is based on the observation that, since more applications come from inventors in high‐income countries, a developing country's patent system is most likely to issue patent rights to foreigners. Therefore, a stronger patent system serves mainly to transfer wealth from domestic consumers to inventors in high‐income countries (Maskus, 2000).

The second argument against patents is related to the first, but focuses on domestic producers, holding that stronger patent regimes in developing countries create difficult conditions for domestic industry to compete against global technological leaders. Under a weaker patent regime, developing‐country firms have greater freedom to imitate technologies invented in wealthier countries. The developing‐country imitators can then move, at lower cost, through the crucial phases of catching up to the global technological frontier. Once they have caught up, it is conceded, IPRs can then be accordingly strengthened. This pattern, it is argued, has been followed repeatedly in history, first by firms in Germany and the United States catching up to those in Britain and France in 19th century, and successively by firms in Japan, Korea, and most recently in China catching up to those in the West.

A third major argument against patents in developing countries has focused on humanitarian issues of access to technologies that meet fundamental human needs, such as food security and essential medicines (Gold & Lam, 2003; Kapczynski, Chaifetz, Katz, & Benkler, 2005; Orsi, Camara, & Coriat, 2006). Stronger patent regimes in developing countries, it is argued, tend to increase prices of food and medicines, particularly for the poorest of consumers for whom these categories make up a large share of typical household budgets. Furthermore, agriculture and health‐care represent some of the most widespread forms of economic activity in developing countries: with large segments of the population deriving their livelihoods from smallholder agriculture and with malnutrition, poverty, and infectious disease creating disproportionate public health and economic burdens. In sub‐Saharan Africa, such conditions certainly are observed, and, for example, high profile objections erupted in South Africa in the late 1990s regarding patents on antiretroviral human immunodeficiency virus drugs (Fisher & Rigamonti, 2005; Ostergard, 1999).

Other scholars caution, however, that developing‐country policymakers should not too‐readily neglect patent systems, given the increasingly important role that knowledge plays in economic growth. Foremost, it is countered, under a weak patent system domestic industry, entrepreneurs, or publicly funded researchers choose not to invest resources in innovative effort, given the lack of incentives from the lack of domestic protections (Chen & Puttitanun, 2005; Krattiger, Mahoney, & Nelsen, 2007). Second, with weaker IPR protections in developing countries, innovators in more developed countries, those who have greater technological capacity to innovate, see fewer incentives to invest resources and efforts in creating technologies that meet the different, and sometimes idiosyncratic, needs of producers or consumers in developing countries (Chen & Puttitanun, 2005; Diwan & Rodrik, 1991). A third argument, and one that was widely advanced in advocating for the TRIPS agreement under the WTO treaty, holds that foreign direct investments, particularly of the sort which is anticipated to result in greater technology transfer, capacity development, and economic growth in the receiving countries, are more likely to be made into those countries with patent systems strong enough to afford the foreign investor with sufficient protections.

Yet, as is often the case with such policy debates, the reality of the situation is more complex. Empirical analysis by Gould and Gruben (1996), Maskus (2000), Chen and Puttitanun (2005), and others, have found that the strength of the IPR systems in developing countries—as measured by IPR indexes such as the ones developed by Rapp and Rozek (1990) or Ginarte and Park (1997)—tend to exhibit a U‐shaped relationship with respect to level of economic development. In practical terms, the tendency for a weak IPR system at lower levels of development is due to low levels of institutional capacity to innovate and to domestic political support for maintaining the freedom to imitate foreign technologies, both by low‐tech firms and by consumers who desire low prices. Strengthening of the IPR system, it is argued, comes about as the economy grows and as domestic political support for protection of inventions intensifies, both from leading domestic firms that begin making inventions of their own, for which they seek protection, and by domestic consumers who increasingly demand higher‐quality products that embody or require higher levels of technology from abroad.

Using 25 years of historical data for 64 countries, Chen and Puttitanun (2005) estimate that an upturn in the strengthening of IPRs begins around a relatively low threshold of US$854 per capita gross domestic product (GDP) (in 1995 dollars). In 1980, only South Africa and a handful of smaller countries in sub‐Saharan Africa—including Namibia, Botswana, Gabon, Mauritius, and the Seychelles—met or surpassed this GDP‐per‐capita threshold. By 2000, the number of countries in sub‐Saharan Africa above that threshold had more than doubled. By 2010, the average per capita GDP across sub‐Saharan Africa as a whole exceeded the threshold (US$1,222 in 2010 dollars). In 2015, the roster of countries with per capita GDP above the estimated threshold (US$1,328 in 2015 dollars) included Kenya, Cameroon, Cote d'Ivoire, Sao Tome and Principe, Ghana, Djibouti, Sudan, Zambia, Nigeria, Angola, Congo, Equatorial Guinea, Cabo Verde, and Swaziland: a set of countries that together accounted for over 82% of Africa's GDP and 46% of the population.

As the single country in sub‐Saharan Africa with middle‐income status, South Africa's firms and scientific institutions have long had capacity to generate inventions and to protect them, both domestically and in foreign jurisdictions (Naidoo, 2010). Now, increasingly other parts of sub‐Saharan Africa are reaching a turning point. As countries across the subcontinent exceed Chen and Puttitanun's (2005) estimated threshold of economic development, innovation capacity emerges, domestic invention begins to increase, and, along with that, domestic utilization of the patent system, if available, grows. Yet, more empirical analysis is needed to ground policy discussions of knowledge creation and utilization in sub‐Saharan Africa.

This analysis provides a set of quantitative benchmarks for how patents—and the inventions they protect—have been playing a role in sub‐Saharan Africa. For the period 1980–2010, we explore overall patent filing trends utilizing summary data from the World Intellectual Property Organization (WIPO, 2016). For the same time period, we also focus on patent filing trends in biological inventions for health and agriculture (e.g., genetic resources, vaccines, microorganisms, seeds, living modified organisms, etc.) which have been particularly controversial in developing countries (Blakeney & Mengistie, 2011; Boettiger, Graff, Pardey, Van Dusen, & Wright, 2004; Castle, 2009; Krattiger et al., 2007), utilizing detailed data from the International Science and Technology Practice and Policy (InSTePP) Global Genetics Patent Database (InSTePP, 2016). These patent data are analyzed to determine how extensively patents are being used to protect inventions in sub‐Saharan Africa, by whom, and in which industries. We explore how invention and filing rates in sub‐Saharan Africa compare with invention and filing rates in other countries and regions around the world. We seek to understand how the patent system is being used to mediate the flow of technology transfers into and out of sub‐Saharan Africa, and even among the countries in sub‐Saharan Africa. We are also curious to understand what types of technologies are being patented in sub‐Saharan Africa and whether the mix of technologies has changed over time. Finally, we are particularly interested in how patenting rates over biological inventions for essentials in health and agriculture compare with overall patenting rates in Africa. From these detailed interrogations of available patent data, we establish what historical practice has been in sub‐Saharan Africa, and we draw a policy implications regarding the patent system and its emerging role in encouraging and enabling future knowledge‐driven economic growth.

## 2 BACKGROUND

### 2.1 Inventions and how they are patented

Initially, when an invention is made, information about that new technology is known only to its inventors. The inventors then—often in close consultation with their employer or legal counsel—make decisions about whether and where to file applications for patent protection. If they choose not to file, then, obviously, information on their invention will not show up in patent data; they may choose instead to keep information about their new technology secret, or they may choose to disclose information about the new technology in a scientific article or technical publication (Hall, Helmers, Rogers, & Sena, 2014). If the inventors do choose to file one or more patent applications, then a rich source of data becomes available, in the form of the primary technical information disclosed in each patent application, and in the form of secondary information that can be derived from the patterns of where and when patent applications were filed, and by whom (Griliches, 1990; Pavitt, 1985).

Most patent jurisdictions are national in scope, with a national patent office that receives and reviews patent applications and issues patents, which are then enforced by the courts within the borders of that nation. In several parts of the world—such as in Europe and in Africa—groups of countries have joined together to create regional patent offices, at least for purposes of receiving and reviewing applications. Yet, even in these cases when patents are issued by a regional office, those patents are accepted within each member country as a national right. An invention is under patent protection in a given country only to the extent that a patent is currently in force in that country.

### 2.2 Patent families

Given this national nature of patent rights and the international rules long‐established under the Paris Convention of 1883, inventors initiate the patenting process by making an initial or *priority* patent application at a patent office of their choosing. Today 175 countries are members of the Paris Convention, including most African countries. Inventors in both member and nonmember countries tend to follow the typical pattern of filing an initial application at the patent office of the country in which they reside and therefore the country in which they made the invention. However, this is not required and is not always the case. If the inventors wish to seek patent protection for their invention in other markets, they can file applications at those other national or regional patent offices, either directly or via the facilitating mechanism of the Patent Cooperation Treaty administered by WIPO (see Viksnins & McCrackin, 2007). If inventors file in multiple patent offices, the follow‐on (or parallel) patent applications reference back to the priority patent application that was made at the office of first filing.

These documents that result from the patenting process for a given invention collectively make up what is known as a *patent family* for that invention: a set of one or more related patent documents at one or more patent offices that all reference back to the same priority application and therefore represent the same underlying invention (EPO, 2017; Martinez, 2011; WIPO, 2017). It should be clear that a patent family, as observed in the patent data, can continue to grow over several years as additional patent applications are filed and published and as new patents are issued for a given invention, potentially in several different patent offices. For the purpose of this analysis, we rely on the systematic identification of patent families in global patent data provided by INPADOC, a public service of the European Patent Office (EPO, 2017).

All patent families can, by definition, be characterized by their *office of first filing*, that patent office at which the initial or priority patent application on the invention was filed. *Domestic* patent families result when protection is sought in just one jurisdiction and thus consist of patent documents from just a single patent office, while *international* patent families result when protection is sought in more than one jurisdiction and consist of patent documents from multiple patent offices. WIPO has adopted the somewhat more general term *foreign‐oriented* patent families, which is defined as a patent family having at least one filing at an office that is different from the office of the applicant's origin, that is, the country of residence of the first‐named applicant on the patent application (WIPO, 2017). Throughout the following analyses, unless otherwise indicated, we consider the *patent family* as the measure of an *invention*.

### 2.3 African patent institutions

A basic understanding of the structure and geographic coverage of the main patent offices in sub‐Saharan Africa is necessary to understand and interpret the data on patenting activity in Africa, as well as to appreciate the institutional responses that have emerged from the formation of African patent policies. While these institutions handle patent filings being made in sub‐Saharan Africa, both by residents and by foreigners, they also influence patent filings made in foreign patent offices by residents of sub‐Saharan Africa. This is due to a general tendency for inventors to make an initial patent application at their own domestic or regional patent office and then, from the basis of that priority application, as a sort of “springboard,” to launch into subsequent foreign filings abroad. Thus, these African institutions dictate the architecture of the patent data and influence the rates of patent filings coming into Africa and going out of Africa.

**Figure 1:**
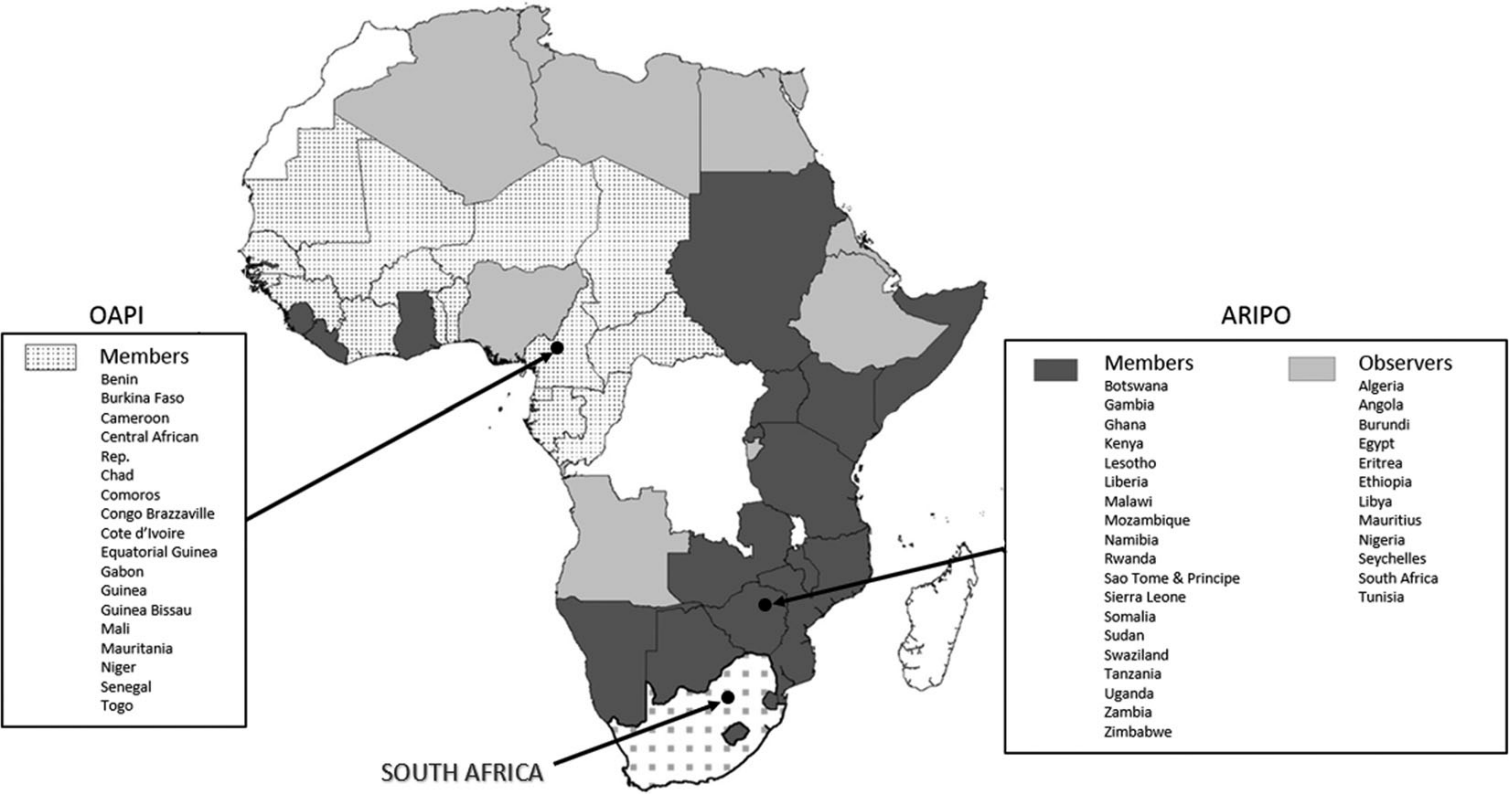
Africa's major patent offices and their members

Three patent offices in sub‐Saharan Africa represent most of the countries and the majority of economic activity of the subcontinent. These include the national patent office of South Africa, in Pretoria (Barratt, Snyman, & Lutchman, 2018; Naidoo, 2010; Pechacek, 2012), and two regional patent offices—the African Regional Intellectual Property Organization (ARIPO), based in Harare, Zimbabwe (Adewopo, 2002; ARIPO, 2016; Nwauche, 2003), and the *Organisation Africaine de la Propriété Intellectuelle* (OAPI), based in Yaounde, Cameroon (Adewopo, 2002; Botoy, 2001; Nwauche, 2003; OAPI, 2015)—for which member countries are shown in Figure 1. The vast majority of patent filings in sub‐Saharan Africa occur at one of these three main offices. South Africa and the member countries of these two regional patent offices encompass 56% of the population and 60% of the economic activity as measured by GDP of sub‐Saharan Africa. Countries with independent national patent offices make up another 37% of GDP of sub‐Saharan Africa. Of these, Nigeria and Angola, which are both ARIPO observer states, represent perhaps the most conspicuous gaps in the patent system in sub‐Saharan Africa. Together these two countries account for 20% of sub‐Saharan Africa's population and fully one‐third of its GDP. Yet, the national patent offices for Nigeria and Angola report just a handful of patent filings to WIPO and thus do not appear to have effective patent systems. The few remaining countries that are entirely unaffiliated with the regional patent offices—such as the Democratic Republic of Congo, Madagascar, and South Sudan—are among the least developed countries and, likewise, account for only a handful of patent filings (For brief histories and more details on Africa's major patent offices, see the Supporting Information Appendix, Section I.)

### 2.4 Patent data sources for sub‐Saharan Africa

To ascertain trends in patenting—both of inventions made by residents of sub‐Saharan Africa and of inventions made elsewhere but filed in one or more of the patent jurisdictions of sub‐Saharan Africa—we rely upon two sources of patent data (see the Supporting Information Appendix, section 2 for greater detail on data sources and methods). WIPO's Statistics Database provides summary statistics of annual counts of patent publications and patent families across all technologies and all patent offices (WIPO, 2016). The WIPO database contains summary data that include annual counts of patent families that originate from each country and annual counts of patent families that are filed at each reporting patent office around the world. The data also include counts of annual patent publications at each reporting office by technology type. These data provide context regarding the full scope of patenting activities in sub‐Saharan Africa. The WIPO Statistics Database reports 173,079 total patent family filings in South Africa, 4,813 total filings in ARIPO, and 7,106 total filings in OAPI from 1980 to 2010.

The second data source is the InSTePP Global Genetics database (InSTePP, 2016), based upon data originally compiled from Thomson Innovation (today Derwent Innovation of Clarivate Analytics), which provides detailed records of selected patent filings related to biological subject matters, including biological research tools, nucleic acids (DNAs and RNAs), proteins, and associated biological materials, such as biologics, biotherapeutics, or biopesticides, as well as genetic resources, breeding materials, and modified living organisms. This database has detailed information on individual patent publications (both applications and granted patents), organized into patent families, and can therefore connect a range of characteristics for each invention, including country of origin, the jurisdictions of all patent family filings, patent assignees, technology classifications, and industry of application, allowing for detailed analyses of invention and filing trends of biological inventions in sub‐Saharan Africa. The InSTePP Global Genetics database identifies 43,696 total biological patent family filings in South Africa (with data coverage for years 1980–2005), 2,161 total biological patent family filings in ARIPO (with data coverage for years 1980–2005), and 2,251 total biological patent family filings in OAPI (with data coverage for years 1980–2002).

Due to data reporting limitations as well as the long lags naturally involved in foreign‐oriented patent family growth and reporting—particularly for filings into smaller patent jurisdictions—an historic window of 1980–2010 was chosen that allows for maximum coverage and overlap between the WIPO and InSTePP data. A major challenge for the analysis of intellectual property in sub‐Saharan Africa is incomplete data reporting (see discussion in Supporting Information Appendix, section 2.2). In addition, for some analyses below the timeframe ends even earlier—in 2003, 2005, or 2008—again due to incomplete data or truncation specific to each variable.

## 3 UNDERSTANDING AFRICA IN A GLOBAL CONTEXT

**Figure 2:**
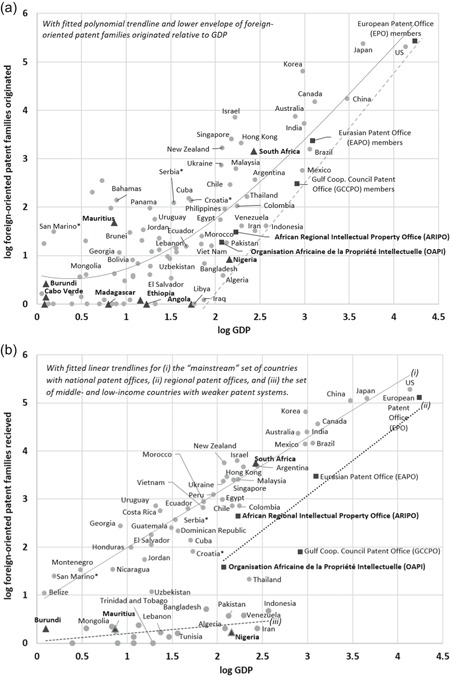
Relationships between economic size and patent family filings, 2004–2008. (a) Foreign‐oriented patent families originated. (b) Foreign‐oriented patent families received. Relationship between GDP and (a) the numbers of foreign‐oriented patent families originated from national and regional filing offices, and (b) the number of foreign‐oriented patent families received at the identified national and regional filing offices. Data represent log of average annual counts of foreign‐oriented patent families originated or received, respectively, over 5 years from 2004 to 2008 (from WIPO) is plotted against log of average national GDP at market prices in billions of current U.S. dollars over the same years (from World Bank). For regional patent offices, inventions originating by country and GDP of all member states are aggregated. Regional patent offices are designated with square symbols. Sub‐Saharan African countries with independent patent offices are designated with triangular symbols. Names of all sub‐Saharan African entities in bold. (a) Members that joined the European Patent Office after 2008 are considered separately from the EPO in this analysis. Following WIPO Statistical Database definitions, the origin of a patent family is defined by the country of residence of the first listed inventor or applicant on the priority application. GDP, gross domestic product; WIPO, World Intellectual Property Organization. *Source*: World Bank (2016) and WIPO Statistics Database (2016)

How do patent activity levels of sub‐Saharan Africa compare with those of other comparable countries or regions around the world? Generally, we expect larger and more developed economies both to generate more inventions internally and to attract more patent filings from abroad. To examine the African economies in a relative context, controlling for market size, Figure 2 plots the count of foreign‐oriented patent families against the GDP of a country (or countries) served by a given patent office. Counting foreign‐oriented patent families provides a somewhat normalized representation of inventions, since the inventions being represented must conform to the patent eligibility policies of at least two jurisdictions and has been considered valuable enough to warrant filing in at least one additional jurisdiction. Figure 2a measures foreign‐oriented patent families originated by country or region, and Figure 2b measures the count of foreign‐oriented patent family filings received by a national or regional patent office. In both panels, we use averages calculated over 5 years from 2004 to 2008, before the impacts of the global financial crisis of 2008–2009 were felt, to smooth out some of the volatilities and idiosyncrasies of annual data. This analysis includes independent national patent offices and regional patent offices. For those countries that are members of regional patent offices, country‐level data are aggregated together and represented as a region, to enable comparison of the patenting activities of those regional offices according to the collective sizes of the markets they represent.

### 3.1 Where foreign‐oriented patent families originate

The numbers of foreign‐oriented patent families originating from within a country or region is related to the overall size of that country's or region's economy, following a double logarithmic trend that appears roughly J‐shaped (Figure 2a). Starting at the high end of the curve, with the largest economies, the United States, Europe, and Japan are also the sources of origin of the largest numbers of foreign‐oriented patent families. These are then followed by a set of other large economies, including Korea, which tracks closely to its neighbor Japan, as well as Canada, Australia, and the so‐called BRICS, consisting of Brazil, Russia (which is represented here as part of the Eurasian Patent Office), India, China, and South Africa. Below these economies, in terms of market size, the variation from (the J shaped) trend in the numbers of inventions originating within countries grow wider. We see a group of small but relatively wealthy countries, including Bermuda, Barbados, Bahamas, and Malta, with relatively more inventions per GDP, causing the upturn that forms the hook of the “J” in the J‐shaped trend. There are certainly many small‐ and mid‐sized economies originating few or no foreign‐oriented patent families. However, above a certain size, all economies are originating foreign‐oriented patent families, with a lower envelope (see dashed line in Figure 2a) defining a minimum number of inventions per GDP that appears to be strongly increasing in GDP.

African countries and regional patent offices (labeled with boldface font in Figure 2a) are found in different segments of this global landscape in terms of originating foreign‐oriented patent families. South Africa clearly fits its characterization as belonging among the BRICS. About the same number of foreign‐oriented patent families were originated in South Africa as in Brazil, even though the GDP of South Africa is significantly smaller than the GDP of Brazil. South Africa accounts for many more inventions than Argentina or Indonesia, economies that are closer to it in size in terms of GDP. The two regional African patent offices, ARIPO and OAPI, originate fewer foreign‐oriented patent families than many comparably sized economies, but they are not altogether outliers. OAPI members, for example, account for a regional economy comparable in GDP to that of Pakistan, and they originate a similar number of foreign‐oriented patent families. Nigeria has a GDP comparable to the GDP of all ARIPO member states combined. Nigeria also accounts for a similar, albeit somewhat lower, number of foreign‐oriented patent families as all ARIPO member states combined. It is notable, however, that most of the foreign‐oriented patent families that originate in Nigeria are not, in fact, being filed in Nigeria; they are, however, being filed elsewhere in the world, particularly in the United States and the United Kingdom. At the lower extreme, there are several sub‐Saharan African countries with independent patent offices that originate essentially no foreign‐oriented patent families, lying along the horizontal axis of Figure 2a. These include Angola, Ethiopia, Madagascar, Cabo Verde, and Burundi. Finally, we note that Mauritius appears to be approaching the club of small island states of Bermuda, Barbados, the Bahamas, Malta, and others. In fact, Mauritius originates a greater number of foreign‐oriented patent families than Nigeria, than all OAPI member states combined, or than all ARIPO member states combined.

### 3.2 Where foreign‐oriented patent families are filed

The numbers of foreign‐oriented patent families that come to be filed in a given country or region is likewise related to the overall size of that country's or region's economy, with a few exceptions, as illustrated in Figure 2b. There are, in fact, three notable trends in the relationship between the logarithm of GDP and the logarithm of the numbers of foreign‐oriented patent families received.

The main trend, representing what may be described as the global “mainstream” of the patent filing system, is an almost linearly increasing relationship, involving the majority of countries, between the log of GDP and the log of foreign‐oriented patent families received. In essence, the larger the economy, the more filings it receives, all else being equal. This is due both to receiving more filings on inventions from abroad, as well as having more domestic inventions, filed at home, that also end up getting filed abroad (and thus being defined as “foreign‐oriented”). This trend is visible in Figure 2b, as the diagonal running from lower left to upper right. In the extreme upper right, Europe, the United States, Japan, and other large economies like Canada and Australia receive the most patent family filings globally. Many middle‐ and low‐income countries also lie along this main trend line, with the smaller economies receiving proportionately fewer foreign‐oriented patent filings.

In terms of count of foreign‐oriented patent family filings received, South Africa lies, in fact, just above this main trend line, aligning almost exactly with Argentina (even though, as seen in Figure 2a, South Africa originates more inventions that Argentina). Israel, Hong Kong, and New Zealand, although somewhat smaller economies than South Africa, receive about the same number of foreign‐oriented patent family filings, which might be expected for countries with relatively higher levels of economic development. The other BRICS, with larger economies than South Africa, receive proportionately larger numbers of foreign‐oriented patent family filings but are also within trend. Brazil, Mexico, and India lie closely together, but they are also very comparable to Australia and Canada. China receives almost as many foreign‐oriented patent family filings as the United States, Europe, or Japan.

The second notable trend in Figure 2b, is that all the regional patent offices lie below the primary trend line, meaning that the regional offices receive comparatively fewer foreign‐oriented patent family filings relative to the aggregate GDP of the economies they serve. This is likely due to several factors. Regional offices are each, to varying degrees, complemented or augmented by filings made at the national patent offices of their member countries. Thus, the count of patent families filed at the regional office likely underrepresents total filings within the region. Because of the nature of the WIPO summary data, it is not possible to determine how many of the foreign‐oriented patent family filings at different offices represent the same inventions. Anticipating that the resulting over count would be a more serious problem, this analysis chose the lesser of possible biases and dropped the counts of filings at the member states' national offices. Second, regional patent offices have tended to be an institutional response of countries with less‐developed and therefore weaker patent systems, which relates to the third observed trend, below. The Eurasian Patent Organization (EAPO) includes the nine member states of the Commonwealth of Independent States, all of which are former Soviet Republics: Russia, Turkmenistan, Belarus, Tajikistan, Kazakhstan, Azerbaijan, Kyrgyzstan, Armenia, and Moldova. Notably, among the former republics of the Soviet Union, Ukraine, and Uzbekistan, as well as the Baltics, are not members of EAPO. The Gulf Cooperation Council Patent Office (GCCPO) is made up of six countries on the Arabian Peninsula: Saudi Arabia, Bahrain, Kuwait, Oman, Qatar, and United Arab Emirates. Among the regional offices, the two African regional offices, ARIPO and OAPI, conform to the observed relationship between size of the markets represented and the number of foreign‐oriented patent families filed. ARIPO is shifted downward from the primary trend line to an extent that is very much comparable to how the European Patent Office is shifted. Even OAPI does not deviate as far from the primary trend line as does the GCCPO, which appears to lie the furthest away.

The third trend involves a diverse set of middle‐ and low‐income countries situated close to the bottom axis, meaning that they receive very few foreign‐originated patent applications relative to other countries with similarly sized economies. This suggests that the patent systems in these countries are weaker than the conditional norm (i.e., after controlling for economy size) and, therefore, are not considered worthwhile offices in which to file for patent protection, all else being equal. However, there may be other factors involved. Many of these are energy rich countries. Others are countries that have experienced civil conflict, or for other reasons have been less integrated with the global economy. Three sub‐Saharan African countries that independently operate their own patent offices—Nigeria, Mauritius, and Burundi—all fall within this cluster. Of these, Mauritius may be considered too small to matter as a destination market, even though it proves to be a source of inventions.

These summary statistics reveal that the patent activity levels of sub‐Saharan African countries—both in terms of inventions they originate and the filings they receive—are comparable to similarly sized countries or regions around the world. The differences among sub‐Saharan African countries are greater than the differences between them and comparable countries around the world. South Africa aligns with global patent activity trends, conditioning on the size of its economy, comparing particularly closely to it BRICS counterparts. The member countries of ARIPO and OAPI generate and receive fewer inventions than comparably sized developed countries, but still perform at a level comparable to the other regional patent offices. Only a handful of African countries with independent patent offices—the largest of which is Nigeria—are relatively uninvolved in patenting activities relative to global trends. Still, given the size of its economy, Nigeria does give rise to inventions being filed abroad. And, the patent activity profile of Mauritius is comparable to other small, higher‐income island states.

## 4 PATENT FILINGS IN SUB‐SAHARAN AFRICA

The comparative assessment in the previous section indicates that the only jurisdictions in sub‐Saharan Africa to receive appreciable annual patent filings are the three main offices of South Africa, ARIPO, and OAPI. Annual filings in all other independent national offices are so small as to barely register. We therefore focus in this section on patent family filing trends in the three main patent offices.

### 4.1 Patent family filing trends

**Figure 3:**
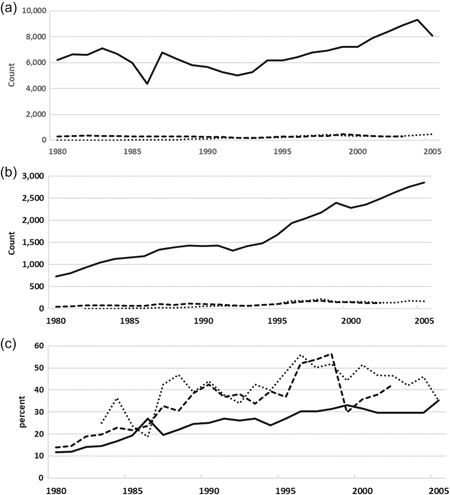
Inventions Filed in sub‐Saharan Africa, 1980–2010. (a) Total inventions filed, by office. (b) Biological inventions filed, by office. (c) Biological inventions as percent of total inventions filed, by office. Data are counts of inventions filed in sub‐Saharan Africa's three main patent offices. (a) Includes total initial filings of patent families, in all technologies, according to WIPO Patent Statistics Database. (b) Includes initial filings of patent families involving biological subject matter inventions, according to the InSTePP Global Genetics Patent Database. (c) Plots the share that biological patents represent of total filings.InSTePP, International Science and Technology Practice and Policy; WIPO, World Intellectual Property Organization. *Source*: WIPO Statistics Database (2016) and InSTePP Global Genetics Database (2016)

Annual counts of the initial filings for patent families in the three main offices of South Africa, ARIPO, and OAPI, are plotted in Figure 3a, 3b, and 3c, respectively. The count of total new patent families includes both domestic‐only and foreign‐oriented patent families, to represent overall filing activities in these jurisdictions (see also Supporting Information Appendix, Table A1).

The first major observation is that filings in South Africa are significantly greater than those in the two regional jurisdictions. In fact, filings in South Africa are greater than in all other sub‐Saharan African jurisdictions combined. In 2000, South Africa's GDP was 55% larger than the combined GDPs of the ARIPO member states and 155% larger than the combined GDPs of the OAPI member states; yet, in that same year, South Africa reported 2,350% more patent family filings than ARIPO (7,206 compared with 307) and 1,790% more than OAPI (7,206 compared with 403) (Figure 3a).

For South Africa, the volatility in filing levels from 1983 through 1992 corresponds to the period of economic sanctions, the end of the apartheid government, and prevailing market uncertainties over the change in government. Annual filing rates began to recover after 1992, as Nelson Mandela's new government proved stable. Starting from very low levels, filing activity at the regional patent offices of ARIPO and OAPI grew, at least modestly, throughout, although OAPI experienced some stagnation during the 1980s. At both ARIPO and OAPI, filings began to increase in the late‐1990s, and by the mid‐2000s both were registering around 500 annually.

Patent family filings for biological inventions (Figure 3b) follow similar trends, dominated by the numbers of patent families filed in South Africa and with much lower levels of filings in the other offices. It appears that innovation in the bioeconomy makes up a significant share of all innovation being pursued and applied in sub‐Saharan Africa. Figure 3c indicates the annual share of patent families on biological subject matter, as identified in the InSTePP Global Genetics Patent Database, out of patent families overall, according to WIPO Statistical Database. According to this, biological inventions filed in South Africa make up about 30% of the total, at least since the mid‐1990s. Similarly, at the OAPI and ARIPO offices the annual count of biologically oriented patent families, according to InSTePP data, make up 40–50% of the annual count of total patent families, according to WIPO data (Figure 3c). This relatively large share may reflect the heavy dependence of these economies on agriculture and natural biological resources. The large proportion of biological inventions may also help to explain the high degree of concern that African policymakers and others have expressed over policies governing the patentability of technologies involving biological subject matters (Taylor & Cayford, 2003).

### 4.2 Technology and industry trends

Of the filings received by patent offices located throughout sub‐Saharan Africa, the kinds of technologies and the industries they serve indicate the nature of the economic activity that is being influenced by patents. It also indicates which industry stakeholders are more likely to seek to influence patent policies.

In analyzing technology and industry trends revealed in patent data, it is crucial to keep in mind that even in high‐income countries, not all technologies are equally amenable to patent protection, and therefore not all industries equally utilize patent protection as a means of appropriating returns on investments in innovation. Particularly high propensities to patent have been observed in pharmaceuticals and chemicals (Levin et al., 1987; Pakes, Simpson, Judd, & Mansfield, 1989). It may be expected that such trends are even more pronounced in low‐income countries with less diversity of industry and generally newer patent systems. Thus, while patent data reveals only a partial picture of knowledge‐driven economic activity, it is helpful in identifying technologies and industries that are actively innovating.

#### 4.2.1 Overall technology categories

WIPO summarizes, for each country, annual counts of patent grants by category of technology, utilizing the International Patent Class (IPC) codes. From these data, we see that in relative terms the broad categories of technologies being protected with patents at the three main patent offices in sub‐Saharan Africa have remained quite stable over the past 30 years (see Supporting Information Appendix, Figure A2). In South Africa the most‐prevalent technology categories have not changed since 1980, with the same ten broad categories collectively representing about 60% of total annual patent grants. At the ARIPO and OAPI offices, the ten most prevalent categories make up between 65% and 75% of annual publications.

Six of the ten top technology categories are common across the three main patent offices in Africa. These six include pharmaceuticals, organic fine chemistry, basic materials chemistry, chemical engineering, biotechnology, and civil engineering (Supporting Information Appendix, Figure A2, comparing A2a, A2b, and A2c). Pharmaceuticals technology is the single largest category at all three offices. At South Africa's patent office, pharmaceuticals make up 10–15% of annual patent publications in the latest years (Supporting Information Appendix, Figure A2‐a). At the ARIPO and OAPI offices, the share of patent publications in pharmaceuticals has increased over time, coming to make up 20–25% of total patent publications in recent years (Supporting Information Appendix, Figure A2b,c). Four categories describing chemistry—organic fine chemistry, basic materials chemistry, chemical engineering, and food chemistry—make up 20–30% of publications at each of the three offices in recent years. Biotechnology is also found among the top 10 categories at all three offices, making up 4–6% of publications in the most recent years at all three offices.

#### 4.2.2 Industries of application for biological inventions: The scope of Africa's bioeconomy

The IPC‐based technology categories reported by WIPO relate to the technical characteristics of an invention. This, however, does do not necessarily identify the industry in which that technology is being applied, despite significant efforts to create concordances mapping patent classes to industries of application (Evenson, Putnam, & Kortum, 1991; Johnson, 2002; Lybbert & Zolas, 2014; Verspagen, van Moergastel, & Slabbers, 1994). The InSTePP Global Genetics Patent Database draws upon Derwent World Patent Index (DWPI) Manual Code designations that enable a categorization of patent families by industry of application. While the IPC‐based WIPO category named “biotechnology” represents only about four to 6% of annual patent grants, the InSTePP database, indicates that 30–40% of inventions filed in sub‐Saharan Africa involve biological subject matters (Figure 3c). The difference is likely due to the much wider definition used by InSTePP of biological innovations, following more expansive definitions of the “bioeconomy” that have been espoused in policy discussions (European Commission, 2012; OECD, 2009; White House, 2012). Presumably, a large number of the patent publications classified by WIPO in pharmaceuticals, organic chemistry, food chemistry, and others are included in the InSTePP data because they are involved with or related to biology.

Of the biological inventions identified in the InSTePP data, fully 80% carried a DWPI Manual Code indicating an application of that invention in the pharmaceuticals industry, while 20% carried a DWPI Manual Code indicating an application in veterinary medicine (Table 1). Most of those indicated as “Veterinary” are also identified as “Pharmaceuticals.” Meanwhile, 17% of biological inventions indicated an application in “Agriculture.” Other industries of application include “Energy” (6%), “Food and beverage manufacturing” (6%), “Paper and textile manufacturing” (4%), and “Environmental conservation and remediation” (4%).

**Table 1:** The industry of application for inventions involving biological subject matter filed at sub‐Saharan African patent offices 1970–2010, according Derwent World Patent Index Manual Code designations

Industry of application	Sub‐Saharan African patent publications involving biological subject matter (count)	Share^a^ (%)
Pharmaceuticals	43,386	80.0
Industrial chemicals	16,926	31.2
Veterinary	11,705	21.5
Agriculture	9,398	17.3
Bioenergy	3,320	6.1
Food and beverage	3,188	5.8
Pulp, paper, and textile	2,275	4.1
Environment	2,165	3.9

*Source:* InSTePP Global Genetics Patent Database (2016).

^a^ Shares out of 54,194 patent publications on inventions involving biological subject matter, filed with patent offices in sub‐Saharan Africa. Shares sum to more than 100% because patent publications are counted in more than one industry of application. See the Supporting Information Appendix for list of DWPI Manual Codes used to designate each industry of application.

### 4.3 Countries of origin

Where do the inventions being filed in sub‐Saharan Africa originate? What share of the inventions filed in sub‐Saharan Africa comes from domestic inventors, from inventors in other countries of Africa, and from inventors outside Africa? Who is making the most use of the patent protections provided by the patent offices of Africa? Do we see evidence of the patent system being used to mediate South‐South technology transfers? To assess country of origin, WIPO's Statistical Database considers the country from the listed address of the first inventor or applicant.

#### 4.3.1 Countries of origin of patent filings in South Africa

**Figure 4:**
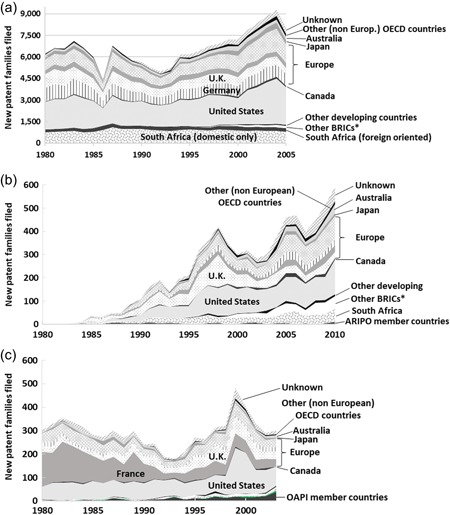
Countries of origin of all patent families filed at the patent offices of South Africa, ARIPO, and OAPI. (a) *South Africa, 1980–2005*. (b) ARIPO, 1980–2010. (c) OAPI, 1980–2003. Other BRICs include Brazil, Russia (including Soviet Union before 1991), India, China (including Hong Kong).ARIPO, African Regional Intellectual Property Organization; OAPI, Organisation Africaine de la Propriété Intellectuelle. *Data Source*: WIPO Statistics Database (2016)

At the national patent office of South Africa, according to summary statistics from WIPO, the greatest share of patents filed are part of patent families that originated in Europe. Europe accounts for about 40% of total patent family filings in South Africa over the 25 years (Figure 4a). Among these, Germany is the largest European source of inventions (12% of total inventions), followed by the United Kingdom (10%) and France (5%). The United States, at about 30% of total patent family filings in South Africa over the 25 years, accounts for fewer patent families than Europe as a whole, but for more than any single European country.

Of the inventions filed in South Africa, 17% originated domestically from within South Africa. While this share may appear modest, it is comparable to the shares of domestic inventions observed in a number of high‐income countries, such as New Zealand, Canada, or Sweden, which receive similarly high proportions of patent filings from abroad. It is not unusual in a smaller economy for domestic inventors to account for a minor percentage of total patent family filings received. The high shares of filings by domestic inventors observed in the larger patent offices, such as the United States, Europe, Japan, and China, are due to a combination of the large size of those economies—thus the relatively large share of world inventions accounted for by domestic inventors—and a “home market bias” in filing propensities demonstrated by inventors everywhere. To illustrate how it might be considered the norm for a national patent office to serve more foreign inventors than domestic, Baumol (1993) relates a thought experiment of an imaginary world made up of just ten countries with equally high income levels, all actively investing in R&D and all trading openly with one another: In such a world, the shares of inventions at each country's patent office would consist, on average, of 10% domestic inventions and 90% foreign inventions.

The number of inventions filed in South Africa by residents of South Africa has remained remarkably stable, at around 1,000 inventions per year from 1980 to 2005 and has not fluctuated nearly as much as the number of inventions being file in South Africa from abroad, which presumably occurred in response to changes in economic and political conditions. In recent years, domestic filings have amounted to about 15% of total filings. In addition, the rate of domestic invention filings by residents of South Africa has also stagnated: the total number of inventions filed in South Africa by South African inventors in 2005 was in fact slightly lower than the number of inventions filed 20 years earlier, in 1985.

A relatively small share of the inventions protected in South Africa come from other developing and emerging economies. The share has grown from about zero as recently as 1995 to about 3% by 2005 (the latest year from which WIPO summary statistics are available). This mostly consists of inventions originating in the other “BRICS” nations—the large emerging economies of Brazil, Russia, India, and China, (often considered together with South Africa) several of which are beginning to invest more heavily in public science and private R&D. The percentage of inventions from other countries of sub‐Saharan Africa protected in South Africa is close to zero, with the absolute number of inventions from other African countries in the single digits.

The distribution of where inventions filed in South Africa originate has remained remarkably stable over a 25‐year period (Figure 4a). While absolute numbers have risen and fallen, the relative shares from each of the countries or regions of origin have not changed significantly over this time. Inventors in South Africa account for about 15%. Inventors in other developing and emerging economies now account for about 3% of the patent families filed in South Africa annually. Inventors in Europe and the United States account for the bulk, at about 70%. Inventors in other high‐income countries account for the remaining 12% of inventions filed in South Africa.

#### 4.3.2 Countries of origin of patent filings at ARIPO

At ARIPO, the total number of inventions being filed is less than one‐tenth of those being filed at South Africa's patent office. Yet, the pattern of countries of origin of inventions being filed displays several similarities with South Africa (Figure 4b). For one, the largest share of patent families at ARIPO, an average of 40% annually, originate from inventors in Europe. Also similarly, the largest single country of origin of patent families at ARIPO is the United States, accounting for an average of 27% of new patent families each year.

Among European countries, the largest source of inventions filed at the ARIPO office is the United Kingdom, accounting for an average of 15% of total inventions filed each year, followed by France (5%) and Germany (4%). This likely reflects the legacy of trade relations and business ownership ties with the United Kingdom for many of the ARIPO member countries, as well as similarities of language and legal systems that reduce the transaction costs of adapting a patent application for a British or European Patent Office filing to become a filing at ARIPO.

Collectively, developing countries account for 17% on average, the same share as observed in South Africa. Almost 10% of the inventions filed at ARIPO each year come from inventors in South Africa, and another 5% of inventions filed come from one of the other BRICs (Brazil, Russia, India, or China). Perhaps the most acute difference between ARIPO and South Africa as an office, is the almost complete absence at ARIPO of “domestic” inventions originating from inventors in ARIPO member countries. Each year less than 10 applications for new inventions come from within the 19 ARIPO member countries while in South Africa a majority of the filings received by the patent office originate from inventors in South Africa (see Supporting Information Appendix, Figure A4).

In summary, inventors in Europe and the United States together account for two thirds, at about 67% annually, of the total number of patent filings. And, inventors in other high income countries account for the remaining 16%. Inventors in developing and emerging economies account for an average of about 17% of the patent families filed at ARIPO annually.

#### 4.3.3 Countries of origin of patent filings at OAPI

At OAPI, annual filings of new patent families (or inventions) are even fewer than those at ARIPO, but there is a longer history. The countries of origin of inventions filed in OAPI (Figure 4c) reflect both OAPI's legacy of close ties with France as well as some of the larger global trends reflected in the origins of patent families filed in South Africa and ARIPO. In the 1980s, a very high share of inventions filed in OAPI—as much as 50% in some years—were by inventors in France. The French share declined by 1990, but still Europeans collectively have continued to account for an average of 47% of inventions filed at OAPI. France has continued to account for fully one‐third (33%). Inventors in the United States have come to account, since 1990, for another 31% of inventions filed at OAPI.

One difference between OAPI and ARIPO is the relatively higher share of resident inventions originating from inventors in OAPI member countries. Resident inventions account for an average of 5% of the inventions filed at OAPI since 1990. Conversely, however, inventions from South Africa account for far fewer, just 1.5%, and, likewise, inventions from the other BRICS (Brazil, Russia, India, China) account for just 1.6%. Inventions from developing and emerging economies overall account for just 10% of patent families filed at OAPI since 1990.

### 4.4 Patent applicants/assignees of filings made in sub‐Saharan Africa

Having established that the bulk of patent filings made in sub‐Saharan Africa originate from countries outside of Africa, with a particularly large share coming from Europe and the United States, we now inquire, more specifically, into what sorts of organizations are utilizing the patent systems in sub‐Saharan Africa. To what extent do the inventions protected in sub‐Saharan Africa come from companies, from individual inventors, or from public sector and academic research institutions?

Under the patent laws of most countries, the rights created by a patent are initially and fundamentally granted to the individual inventor(s) who created the invention. In the process of applying for the patent, each individual inventor then has an option to transfer or assign those rights to another legal entity, typically to the company or other organization that employed the inventor. In most situations, this transfer or assignment of patent rights is governed by terms of the contract between employer and employee, although in some countries the rights and obligations for employee's assignment of inventions to employers is governed by statute (Graff, 2007).

The WIPO database, summarizes patent statistics at a national level, which, unfortunately, is not practical for summarizing the characteristics or locations of the organizations described as “applicants.” The InSTePP Global Genetics Patent Database, however, contains detailed information at the level of individual patent publication records, including the name and address of the applicant or assignee organization(s). These can then be aggregated by patent family. When applicant/assignee data are not provided for a filing in an African jurisdiction, the identity of the applicant/assignee is imputed from other publications in the same patent family, such as those filed in Europe or the United States, offices which regularly do provide applicant/assignee information.

From the InSTePP Global Genetics Patent Database, we found 104,443 designations of an applicant or assignee on the 54,194 patent families filed in sub‐Saharan Africa, with a mean of 1.92 applicants/assignees per patent family. Of these, the dominant share, at 88.2%, were companies. An additional 6.4% were identified to be public sector entities—including government agencies or laboratories, academic institutions, and nonprofit research foundations or hospitals. The remaining 5.4% indicated only the names of the individual inventors, meaning either that a designation of assignment to an employer organization had not yet been submitted or processed at the time of publication or that those individuals were working independently of any such employer (see Supporting Information Appendix, Figure A3).

Tallying up the leading applicant/assignee organizations accounting for patenting activity in sub‐Saharan Africa, out of the 104,443 designations of an applicant or assignee, the identities of the top 20 (listed in Table 2) reflect the leading countries of origin and the leading technologies and industries identified in the previous sections of our analysis. More than half of the top 20 applicant/assignees, including all of the first nine, are global pharmaceutical corporations. At least five of the top 20 are multinational chemical corporations (Akzo Nobel, Bayer, BASF, Dow, and DuPont). Two are multinational corporations focused on consumer and health‐care products (Unilever and Procter & Gamble). Only one of the top 20 is an African organization (the public sector Council of Scientific and Industrial Research, of South Africa).

**Table 2:** Top 20 patent applicant/assignee organizations by count of patent publications on inventions involving biological subject matter filed at sub‐Saharan African patent offices 1970–2010

Rank	Assignee/applicant name	Headquarters	Sub‐Saharan African patent publications on inventions involving biological subject matter (count)
1.	Pfizer	New York, NY, USA	3,995
2.	SanofiAventis	Paris, France	3,239
3.	GlaxoSmithKline	London, UK	2,434
4.	Merck	Newark, NJ, USA	2,203
5.	Roche	Basel, Switzerland	2,159
6.	Novartis	Basel, Switzerland	2,133
7.	Bayer	Cologne, Germany	2,071
8.	AstraZeneca	London, UK	1,385
9.	Johnson & Johnson	New Brunswick, NJ, USA	1,245
10.	DuPont	Wilmington, DE, USA	1,054
11.	BASF	Mannheim, Germany	992
12.	Unilever	London, UK	972
13.	Akzo Nobel	Amsterdam, The Netherlands	955
14.	Eli Lilly	Indianapolis, IN, USA	919
15.	Bristol‐Myers Squibb	New York, NY, USA	914
16.	Dow	Midland, MI, USA	864
17.	Boehringer	Frankfurt, Germany	834
18.	Council of Scientific and Industrial Research (CSIR)	Pretoria, South Africa	667
19.	Procter & Gamble	Cincinnati, OH, USA	609
20.	Abbott Laboratories	Chicago, IL, USA	529

*Data Source:* InSTePP Global Genetics Patent Database.

## 5 INVENTIONS BY SUB‐SAHARAN AFRICANS

Sub‐Saharan African inventors have made only a small contribution to invention and patenting rates globally, both overall and specifically in biological subject matters. This is not surprising, given the levels of economic development in sub‐Saharan Africa (see Figure 2a). However, to understand the nascent knowledge‐creation capacities of sub‐Saharan Africa and prospects for knowledge‐driven economic development, it is helpful to analyze in more detail the nature and sources of those few patented inventions that have been observed.

**Figure 5:**
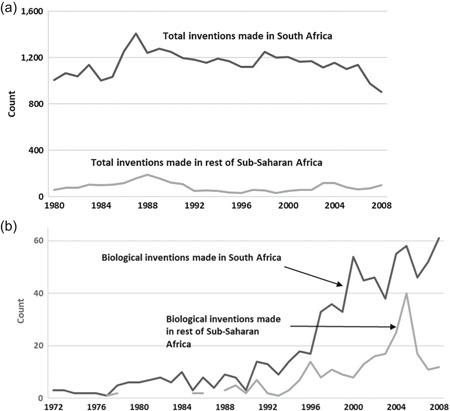
Inventions made in Africa. (a) Total inventions made in Africa. (b) Biological inventions made in Africa. Panel a plots new patent families for which the first inventor or first applicant listed is located in sub‐Saharan Africa. (b) Plots new patent families in biological subject matters with any inventor residing in sub‐Saharan Africa. *Data Sources*: WIPO Statistics Database (a) and InSTePP Global Genetics Database (b)

Patent families originating from the countries of sub‐Saharan Africa through 2010 are shown for all technologies in Figure 5a and for just biological inventions in Figure 5b. Figure 5a shows that overall invention rates in South Africa and in the other countries of sub‐Saharan Africa have not grown significantly since the 1980s. These have held to around 1,000–1,200 inventions per year by inventors in South Africa and about 100 inventions per year by inventors across the rest of sub‐Saharan Africa.

The situation, however, is somewhat different with respect to patenting in genetics and the biosciences (Figure 5b). Biological inventions account for a small share of overall inventions made in Africa—roughly 5% in South Africa and 2% in the rest of sub‐Saharan Africa (comparing levels in Figure 5b vs. Figure 5a). The share has been growing, however, beginning in the early 1990s and continuing through the end of the available data series in 2008.

A cumulative tally is provided in Table 3 that counts how many inventions (patent families) have originated from the countries of sub‐Saharan Africa through 2010—both overall and just in biological subject matters. The cumulative total of inventions by South African inventors over the time period of this analysis is 34,276, according to WIPO data, far greater than the number of patented inventions from the rest of Africa combined. Of that total, just 755 (or 2%) involve biological subject matters, according to InSTePP data. Of the biological inventions that originated in South Africa, 649, or 86%, were assigned to a South African entity. The remaining 14% were presumably invented by residents of South Africa employed by companies with operations in South Africa but based outside South Africa.

**Table 3:** Inventions made and owned by residents of sub‐Saharan Africa

Country of invention	Total inventions, 1980–2010 (WIPO) (count)	Biological inventions, 1970–2010 (InSTePP) (count)	Biological inventions assigned to a domestic organization or firm, 1970–2010 (InSTePP) (count)	Share of biological inventions assigned to a domestic organization or firm (%)
***South Africa***	***34,276***	***755***	***649***	***86***
**ARIPO member countries**				
Botswana	15	0	0	–
Gambia	1	9	3	33
Ghana	16	22	6	27
Kenya	116	46	29	63
Lesotho	5	0	0	–
Liberia	51	2	2	100
Malawi	17	1	0	0
Mozambique	1	0	0	–
Namibia	79	6	6	100
Rwanda	2	0	0	–
Sao Tome and Principe	889	0	0	–
Sierra Leone	44	10	5	50
Sudan	38	0	0	–
Swaziland	7	0	0	–
Uganda	8	6	3	50
Tanzania	7	7	1	14
Zambia	44	4	2	50
Zimbabwe	261	9	7	78
***ARIPO members overall***	***1,601***	***126***	***81***	***64***
**OAPI member countries**				
Benin	28	3	3	100
Burkina Faso	12	3	1	33
Cameroon	89	19	11	58
Central African Republic	5	0	0	–
Chad	7	2	0	0
Comoros	4	1	1	100
Congo	31	3	0	0
Côte d'Ivoire	82	6	1	17
Equatorial Guinea	3	0	0	–
Gabon	24	4	3	75
Guinea	57	3	0	0
Mali	29	1	0	0
Mauritania	28	1	0	0
Niger	30	0	0	–
Senegal	71	14	7	50
Togo	23	0	0	–
***OAPI members overall***	***522***	***56***	***31***	***55***
**Other countries**				
Angola	2	1	0	0
Burundi	19	1	1	100
Cabo Verde	16	1	1	100
Djibouti	0	3	1	33
Eritrea	5	0	0	–
Ethiopia	8	9	1	11
Madagascar	14	11	8	73
Mauritius	224	7	7	100
Nigeria	73	30	11	37
Seychelles	120	6	5	83
***Other countries overall***	***365***	***60***	***40***	***67***

Abbreviation: InSTePP, International Science and Technology Practice and Policy.

*Sources:* WIPO Statistics Database and InSTePP Global Genetics Patent Database.

Collectively, inventors in the largely Anglophone member countries of the ARIPO made and sought to patent just 1,601 inventions overall from 1980 to 2010, according to WIPO data. Of these inventions, 126 (about 8% of total inventions) were specifically in the fields of genetics or biology, according to InSTePP data. And, of those, 81 (64%) were assigned to domestic entities within the ARIPO countries (Table 3). Collectively, inventors residing in the largely Francophone member countries of OAPI made and sought to patent 522 inventions overall between 1980 and 2010 (although WIPO's data coverage for OAPI drops off after 2006). Just 56 inventions involved biological subject matter (equivalent to 11% of total inventions) according to InSTePP data. Thirty one (55%) of these biological inventions went to applicants or assignee organizations based in an OAPI member country (Table 3). Finally, inventors in other countries that are not members of either regional patent office made and filed for protection on 365 inventions over the three decades to 2010, according to WIPO data. We find 60 inventions from these countries in the InSTePP data involving genetics or biology (16% of total inventions) and, of these, 40 (or 67%) went to applicant or assignee organizations based in the inventors' home country (Table 3). Yet, the cumulative shares of African assignees at the bottom of Table 3 misses an important trend: the number of African assignees of biological inventions has grown from virtually none as recently as the mid‐1990s, both in South Africa and in the rest of sub‐Saharan Africa (Supporting Information Appendix, Figure A6).

Only a very small share globally of inventions has come from in Africa. Of the global total of 1,093,038 genetic or biological inventions made between 1970 and 2010, identified in the InSTePP Global Genetics database, only 997, or about 0.1%, were made by residents of sub‐Saharan African; three quarters of these were made in South Africa, and one quarter, or roughly 250 inventions originated in the rest of the countries of sub‐Saharan Africa. In contrast, 228,882 or 23% of the world's patented biological inventions were made by residents of the United States. Yet, for those inventions made in sub‐Saharan Africa, foreign patent filings are quite global. While the majority of South African inventions (see Supporting Information Appendix, Figure A4) are filed only in South Africa, a reasonable share are also filed abroad. In fact, there is a small share of inventions made by South Africans that are only filed abroad and never filed in South Africa.

In tracking individual filings on inventions made by South Africans (Supporting Information Appendix, Figure A5‐a), we see that over one‐third of patents sought by South Africans are in developing and emerging economies. Before 1995, South African inventors made a small number of filings in other independent national offices in sub‐Saharan Africa, but that practice appeared to end with the end of the apartheid government and/or the adoption of the TRIPS agreement in the 1990s. A small number of filings by South African inventors have also been made annually in ARIPO since the 1990s, but virtually none in OAPI. The share of South African inventions being filed in the other BRICS has grown steadily since the 1990s. In the 1980s and 1990s almost half of filings made by South African inventors were in Europe. But, the share being filed in Europe has decreased overall, shifting to focus more on the European Patent Office (EPO) and away from national offices. The share of filings in the United States, Canada, and other OECD countries have remained relatively stable over the three decades analyzed in Figure A5‐a (see Supporting Information Appendix).

Invention from the rest of sub‐Saharan Africa (Supporting Information Appendix, Figure A5‐b) saw a relative surge of activity in the 1980s, although the absolute numbers were quite small. A couple phenomena that arguably account for that trend include extensive national office filings in Zimbabwe and Zambia as well as several hundreds of inventions that originated from Sao Tome and Principe but were filed first at the national patent office in Austria. This anomalous pattern could be attributed even to a single firm or even a single highly prolific inventor in Sao Tome and Principe with a specific connection to Austria. Since 1992, invention rates across the rest of sub‐Saharan Africa have resulted in <100 filings per year, although the patent families resulting from these African inventions show they have been filed quite broadly. About half of these patent filings have been in developing or emerging countries: whether at ARIPO or OAPI, in other developing countries, or in the BRICS. The other half have been filed in high‐income countries (Supporting Information Appendix, Figure A5‐b).

## 6 SUMMARY AND CONCLUSIONS

This investigation of the patenting landscape in sub‐Saharan Africa gives some empirical grounding to discussions of the role of patents in Africa. Even as the subcontinent has experienced steady economic growth, especially since 2000, the rates of domestic inventions that utilize the patent system show evidence of having only begun to increase modestly. To the extent that patents are playing a role in the development of sub‐Saharan Africa, it appears that the first order effect is largely one of facilitating transfers of technology into Africa from abroad. Yet, within this larger picture, there is significant variation in how extensively patents are being used to protect inventions in sub‐Saharan Africa.

In South Africa, both foreign and domestic filings are quite extensive, and very much on par with global trends, given the size of the South African economy. In the regional patent offices of ARIPO and OAPI, both domestic and foreign filings are detectible, although at much lower levels. Still, they are comparable with what is seen in other economies of comparable sizes and levels of development around the world. It is the complete absence of patent activity observed in some of the larger economies—such as Nigeria and Angola—that is more conspicuous. While we see a small but steady stream of inventions from Nigeria, they are only patented in foreign jurisdictions like the United States or Europe, not at home in Nigeria.

Over the three decades covered by this analysis, filings in all three of the main offices in sub‐Saharan Africa have grown, particularly since the mid‐1990s. Yet, rates of patented inventions made by residents of sub‐Saharan Africa have, at best, only held steady over this time period. Although data for more recent years is not as reliable, and there are hints in the data, such as the increasing numbers of sub‐Saharan African assignees, that there may be an upturn occurring in African innovation in the most recent decade. About 1,200 patent families per year have been originating from inventors in South Africa, with the majority of these inventions only filed domestically in South Africa's patent office. Another 100 inventions per year originate from inventors throughout the rest of sub‐Saharan Africa, although interestingly with patent family filings made quite widely around the world. Altogether, when controlling for size of economies, both the invention and filing rates in sub‐Saharan Africa are comparable to those in other national and regional patent offices around the world.

The patterns of patent filings indicate that the main use of the patent system in sub‐Saharan Africa thus far has been for the transfer of technologies into sub‐Saharan Africa. The inventions being patented in sub‐Saharan Africa primarily originate in Europe, the United States, and other OECD countries such as Japan, Canada, and Australia. The top applicants of biological inventions are almost all multinational pharmaceutical, chemical, or consumer‐goods corporations based in Europe and the United States. Still, public sector research organizations account for 6.4% of the biological inventions filed in sub‐Saharan Africa, and South Africa's public‐sector Council of Scientific and Industrial Research is one of the top 20 applicants for biological inventions filed in sub‐Saharan Africa. Thus, the patent system may be beginning to play a role in encouraging commercially relevant innovation from the domestic research capacity in sub‐Saharan Africa which is predominantly public sector.

Yet, a secondary effect of the patent system may be to facilitate the transfer of new knowledge within and among the countries in sub‐Saharan Africa, and between sub‐Saharan Africa and other developing countries in what may be considered “South‐South” technology transfer. About 10% of the patent families filed at ARIPO each year are from South Africa, although there does not seem to be a similar linkage between South Africa and OAPI. Another 10% of the patent families filed at ARIPO each year are from the other BRICS and other developing countries. South Africa's inventors make as many new filings in other BRICS as they do at home in South Africa each year. Half of the filings by inventors in other sub‐Saharan countries are in developing countries or the BRICS.

The types of technologies being protected in sub‐Saharan Africa are those that tend to be most amenable to patent protections, including pharmaceuticals, chemicals, biotechnology, and some engineering. These categories are consistent with that observed in higher‐income countries.

From these analyses, we can draw several policy relevant implications regarding the patent systems and knowledge‐driven economic growth in sub‐Saharan Africa:

- The balance of intellectual trade between sub‐Saharan Africa and the rest of the world is clearly one of net imports of technology to Africa.
- However, the patent system is providing opportunities for inventors in sub‐Saharan Africa to protect their inventions in both other developing countries, in large emerging economies, and even in high‐income economies.
- To some extent “South‐South” knowledge flows are beginning to be mediated by the patent systems of the respective developing and emerging economies involved.
- The types of technologies that tend to be patented in sub‐Saharan Africa are the same types that tend to be patented in high‐income countries: those technologies with higher patenting propensity as a means of appropriating returns to invention. This lends us to advance a testable hypothesis for future research, that foreign filings into sub‐Saharan Africa are part of families larger than average, and thus sub‐Saharan African jurisdictions tend to play host to fewer yet stronger patents than their higher income peers.
- The practical reality of knowledge flows into and out of sub‐Saharan Africa tend to go through “knowledge gateway” countries, with both the legal and commercial capacity to facilitate economic exchanges with the global economic mainstream. The most important of these is South Africa, but other secondary gateways, such as Mauritius, are observed as well.
- While invention and patent filing within Africa are nascent, both data reporting and formal institutional capacities may be lagging relative to actual levels of innovation activity within the economies of Africa.

## CONFLICT OF INTERESTS

The authors declare that there are no conflict of interests.

## Supporting information

Supporting information
