# Supplementary material for: Inventions and patenting in Africa: Empirical trends from 1970 to 2010
Source: J World Intellect Prop. 2019 Nov 25;23(1-2):40–64. doi: 10.1111/jwip.12139 (PMC7078756; doi:10.1111/jwip.12139)
Supplement: Supplementary file 1 — Supporting information [file JWIP-23-40-s001.pdf]

APPENDIX

Online supplementary data and materials for the manuscript:

Inventions and patenting in Africa: Empirical trends from 1970 to 2010

Gregory D. Graff[1,2,3] and Philip G. Pardey[4,5]

1. Department of Agricultural and Resource Economics, Colorado State University, Fort Collins, Colorado, United States
2. Fellow, International Science & Technology Practice & Policy (InSTePP), University of Minnesota, St. Paul, Minnesota, United States
3. Visiting Researcher, Department of Technology Management and Economics, Chalmers University of Technology, Gothenburg, Sweden
4. Department of Applied Economics, University of Minnesota, St. Paul, Minnesota, United States
5. Director, International Science & Technology Practice & Policy (InSTePP), University of Minnesota, St. Paul, Minnesota, United States

Corresponding author: Dr. Gregory D. Graff, B328 Andrew G. Clark Building, 1200 Center Avenue Mall, Colorado State University, Fort Collins, CO 80523-1172, 970-491-4028, gregory.graff@colostate.edu

Acknowledgements: The authors sincerely thank Devon Phillips and Connie Chan-Kang for capable research assistance, and Mats Lundqvist for advice. This analysis arose as a convergence of work under the HarvestChoice project supported by the Gates Foundation and the project "International Assessments of Patenting in Genetics" supported by grant number 5 R01 HG004041-03 from the US National Institutes of Health.

Key words — Intellectual property rights; patent families; patent offices; Africa; international technology transfer

© Copyright 2019 by the authors

1. AFRICAN PATENT OFFICES

1.1 South Africa

South Africa has the oldest patent regime in sub-Saharan Africa, with its early beginnings in the patent laws of several of the predecessor states that eventually came to make up the Union of South Africa. The Cape Colony passed its "Cape Patents Act" in 1860, and Natal passed a patents act in 1870, both of which closely followed English patent law. The first patent legislation in the Union of South Africa was adopted in 1917, and it was largely based on the British Patents Act of 1907. Major revisions were made subsequently by patent acts in 1952, 1978, and 1997. South Africa joined the Paris Convention Treaty in 1947. South Africa was also a founding signatory nation to the WTO Treaty, including the TRIPS Agreement, in 1995. South Africa's patent office today is known as the Companies and Intellectual Property Commission (CIPC), based in Pretoria (Figure 1). For more information see Adewopo (2002), Naidoo (2010) and Pechacek (2012), Barratt et al (2017).

1.2 African Regional Intellectual Property Organisation (ARIPO)

The African Regional Intellectual Property Organisation (ARIPO) was established in Lusaka, Zambia, in 1976 and is primarily composed of Anglophone African countries with legal systems based upon a legacy of British law. ARIPO's formation was a result of joint efforts by the World Intellectual Property Organization (WIPO), the Economic Commission for Africa, and the Organization for African Unity (Adewopo, 2002). In many countries of Anglophone Africa, British patent laws continued essentially unchanged after independence, and, during its early years, ARIPO served as a mechanism to co-ordinate pre-patent grant proceedings among them. Today, ARIPO itself grants patents as well, but each member country has the right to determine if an ARIPO patent will apply within its borders. Thus, patent sovereignty ultimately resides with each member country, and validity and enforcement decisions by the courts of each member country prevail only within the borders of that member country. As of 2017, ARIPO had 19 full members. Most are former British colonies, but not all: Mozambique and Rwanda, for

example, are full members. In addition, there are 12 observer countries, including Nigeria and South Africa, as well as Egypt and several other countries of North Africa. The head office of ARIPO is located in Harare, Zimbabwe (Figure 1). For more information see Adewopo (2002), Nwauche (2003), ARIPO (2016).

1.3 Organisation Africaine de la Propriété Intellectuelle (OAPI)

The Organisation Africaine de la Propriété Intellectuelle (OAPI), also known in English as the African Intellectual Property Organization, was established by collaboration among the countries of Francophone Africa and France's national patent office, the Institut National de la Propriété Industrielle (INPI). OAPI was brought into existence by the Bangui Agreement of 1977, but it was, in effect, a revision and continuation of a previous office called the Office Africain et Malgache de Propriété Industrielle (OAMPI) formed in 1962. Ratification of the Bangui Agreement established OAPI by national law in each member country, and OAPI effectively replaced the French patent office's role in these countries. As a result, OAPI is effectively the sole patent office for each of its member countries. An OAPI granted patent is automatically in force in all member countries; and, moreover, a patent enforcement decision by a court in any one OAPI member country is binding in all (OAPI, 2015). OAPI had 17 member countries in 2017, and its headquarters office is located in Yaoundé, Cameroon (Figure 1). For more information see Botoy, (2001), Adewopo (2002), Nwauche (2003), OAPI (2015).

1.4 Other independent national patent offices

The ARIPO observer states with independent national patent offices make up another 37 percent of GDP of sub-Saharan Africa. Nigeria and Angola, which are both ARIPO observer states, represent perhaps the most conspicuous gaps in the patent system in sub-Saharan Africa. Together these two countries account for 20 percent of sub-Saharan Africa's population and fully one third of its GDP. Yet, the national patent office for Nigeria—the Trademarks, Patents, and Designs Registry—reports to WIPO that it registered only nine patents over the entire decade from 2000 to 2010, and the national patent office

for Angola—the Angolan Institute of Industrial Property—appears to have registered just one patent.1 These two large regional markets thus do not appear to have effective patent systems. The few remaining countries that are entirely unaffiliated with the regional patent offices, such as the Democratic Republic of Congo, Madagascar, and South Sudan, are among the least developed countries of the subcontinent and, likewise, account for only a small handful of patent filings.

2. DATA SOURCES AND METHODS

We seek to ascertain trends in patenting, both of inventions made by residents of Sub-Saharan Africa and inventions made elsewhere but filed in one or more of sub-Saharan Africa's patent jurisdictions. To do so, we rely upon two sources of patent data: WIPO and InSTePP.

2.1 Patent data sources for sub-Saharan Africa: the WIPO and InSTePP databases

The World Intellectual Property Organization (WIPO)'s Statistics Database provides summary statistics of annual counts of both patent publications and patent families across all technologies and all countries or jurisdictions (including ARIPO and OAPI). It is also possible to ascertain counts of publications by technology type and, for most patent families, by country of origin of the invention. These data give important context regarding the full scope of patenting activities in sub-Saharan African countries, but it does lacks detail necessary for some kinds of analyses.

All of the data summaries employed for this analysis come from the patents section of the database.

This analysis draws upon the following patent indicators within the database:

- Indicator 5 – Patent grants by technology

[1] Patent families by origin and first filing office, Total count by filing office, 1980-2010, assessing entries for Nigeria and for Angola. Source: WIPO Statistics Database (http://ipstats.wipo.int/ipstatv2/index.htm?tab=patent). Accessed Dec 2015. For Nigeria, reported patenting has picked up a little in more recent years, with 64 filings in 2011, 42 in 2012, and 50 in 2013 (www.wipo.int/ipstats/en/statistics/country_profile/profile.jsp?code=NG, accessed August 2017). As of August 2017, WIPO reports no further filings in Angola since 2010 (www.wipo.int/ipstats/en/statistics/country_profile/profile.jsp?code=AO)

        - Report type: Total count by filing office
    - Indicator 6 – Patent family by origin and first filing office
        - Reporting type: Total count by (first) filing office
        - Reporting type: Total count by applicant's origin (equivalent count)
        - Reporting type: Count by (first) filing office and applicant's origin
    - Indicator 7 – Foreign-oriented patent family by origin and destination office
        - Reporting type: Total count by (destination) filing office
        - Reporting type: Total count by applicant's origin (equivalent count)
        - Reporting type: Count by (destination) filing office and applicant's origin
    - Indicator 8 – Foreign-oriented patent family by origin and first filing office
        - Reporting type: Total count by (first) filing office
        - Reporting type: Total count by applicant's origin (equivalent count)
        - Reporting type: Count by (first) filing office and applicant's origin

The second data source, developed at the International Science and Technology Policy and Practice (InSTePP) center at the University of Minnesota, based upon data drawn from Thomson Innovation, is a global database of patent filings that protect innovations in genetics, genetic resources, and associated fields of biology and biotechnology. This database had been constructed by exhaustively searching all full-text patent publications for those that claim, describe, or otherwise include reference to nucleotide sequences or peptide sequences. From these, 1,315 International Patent Class (IPC) codes were identified as those most likely to include biological sequence based inventions. These IPCs were then used to download all available patent collections worldwide. The database includes over 5 million individual patent publication records, organized into about one million patent families, and gives details, by invention, regarding country of origin, the pattern of foreign filings, patent assignees, technology type, and industry of application, allowing for detailed analyses of filing trends in Sub-Saharan Africa.

The subject matter of the InSTePP Global Genetics database encompasses all aspects of genetics and biology in human health and medicine, veterinary medicine, agriculture, natural resources, bioenergy, and other applications of genetic or biological innovation throughout the life sciences. The dataset fully encompasses innovation in what is often described as the “bioeconomy” (OECD 2009; The White House, 2012; European Commission, 2012). Not only do such biological innovations meet some of the most fundamental of human needs, including food security and essential health care, but they often also build upon the comparative economic advantages of the low income economies of sub-Saharan Africa, such as developing genetic or biological resources, improving agricultural productivity, or creating opportunities for non-farm rural economic growth in the food and beverage or renewable resource industries. For these reasons, the patenting of genetics and biological resources has been at the center of controversy regarding intellectual property policy since at least the high profile global debates over the Convention of Biodiversity and the TRIPS Agreement in the 1990s (Boettiger et al, 2005).

For this analysis, the InSTePP Global Genetics database was queried for all patent families (inventions) that either (1) with at least one patent-family member document filed in a Sub-Sahara African patent jurisdiction or (2) originated in Sub-Saharan Africa, with at least one listed inventor or assignee located in a Sub-Sahara African country. A total of 54,194 patent families were identified, but only about 1000, or slightly more than 2 percent of these originated in Sub-Saharan Africa.

2.2 Patent data coverage and quality issues for sub-Saharan African jurisdictions

A major challenge for the analysis of intellectual property in sub-Saharan Africa is the incomplete data availability for patent filings. This is an important aspect that must be taken into account when using African patent data. The mechanisms involved in generating patent data available for analysis involve much more than the actual innovation created. The changing, and sometimes unstable, political climate of the countries will affect data availability and intellectual property enforcement alike, and would prove impossible to disentangle these two effects without intense primary research in each specific country.

To illustrate the challenge, in the publications data available from WIPO, the patent office of Botswana had 18 patent applications filed in 1980, 34 in 1981, and 29 in 1982, but does not report any filings after 1982. Likewise, Nigeria reported 441 patent applications in 1985 and 258 in 1990, but none in any other year. It is not clear in cases such as these if the zero counts are a result of a lack of reporting or if, in fact, no filings were made in those years. Fortunately, the major jurisdictions of South Africa, ARIPO, and OAPI have consistently reported their filings to international database, at least from 1990 onwards. WIPO does report application counts (but not grants) for OAPI after 2006. However, there is there is no other national patent office in Sub-Saharan Africa for which there is consistent reporting to WIPO or others.

Figure A1. Coverage of patent data in the InSTePP Global Genetics patent database for the three major sub-Saharan Africa patent jurisdictions and selected national offices

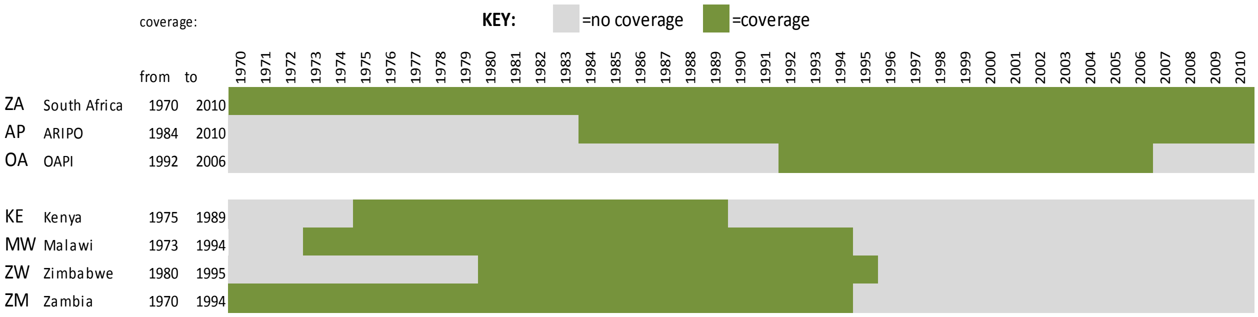

Source: InSTePP Global Genetics Database, 2016, based on Thomson Innovation, 2012

The InSTePP Global Genetics Patent database covers genetics and biological inventions filed in all available jurisdictions worldwide from 1970 to 2010, compiled from Thomson Innovation during the course of the year in 2012. Thomson Innovation draws upon multiple data sources to populate its collection. But it is subject to many of the same data-reporting limitations as is WIPO. Thomson provides publication level data (in contrast to WIPO's country level counts of publications), and thus the standard of reporting for searchable data is higher. Therefore, in the compiled InSTePP Global Genetics Patent

database includes only a few jurisdictions from sub Saharan Africa, including the three main jurisdictions of ARIPO, OAPI, and South Africa, as well as the national offices of Kenya, Malawi, Zambia, and Zimbabwe, although periods of data availability for these national offices are limited (See Figure A1). The InSTePP data has no records for OAPI after 2006. The InSTePP data has records from South Africa from 1970-2010, ARIPO from 1984 to 2010, and OAPI from 1992 to 2006.

Challenges of missing data are partly overcome by shifting the level of analysis from the individual patent application or grant to the *patent family*, the set of all patent records associated with the same invention, including applications and grants at any patent office.

2.3 Measuring individual patent publications versus patent families

One of the complexities of using patent data as an indicator of innovation is the relationship between the number of (observed) patent documents and the number of (unobserved) underlying inventions. In the scholarly literature, it has been typical for studies using patent statistics to count individual patent applications, granted patents, or a combination of corresponding patent applications with their granted patents within a given country (such as the United States) or patent jurisdiction (such as the European Patent Office). Often, in international comparisons, simply because of data availability, it is easiest to count one type of document in different jurisdictions, and then compare those quantities, ignoring the host of legal and technical factors that make those comparisons tenuous: Jurisdictions vary in what types of documents are available and over what time frames; they vary in filing propensity by applicants, by definitions of what constitutes a single invention, by publication lags, and more. As a result, the number of patent documents does not have a simple relationship to the number of inventions, in any given sampling. This is true regardless of whether the sample consists of grants, published applications, or a combination of the two, and whether the sample is drawn from a single patent jurisdiction or many. Instead, the concept of the patent family refers to the entire set of patent documents—including both applications and grants in one or more jurisdictions—that all cover the same invention (or, in some

cases, closely related inventions). For a comprehensive listing of commonly observed configurations of patent families, see Martinez (2011).

Despite the various asymmetries and irregularities of configuration of patent families, and the potential complications that arise from those irregularities, there are several main advantages to using patent families, rather than patent publications, as discrete measures of inventions. First, counting the number of patent families, rather than publications, reduces multi-counting when looking across different countries or jurisdictions, and it therefore provides a better base measure of innovation. One invention can end up generating several different patent applications and grants in different countries. Second, grouping related patent documents into families, particularly across countries or jurisdictions, provides valuable contextual data based upon the structure and dynamics of formation of patent families (as each family "member" carries its own filing and publication dates). It is possible to ascertain in which jurisdictions inventions are first filed, and which lead to subsequent patenting in other jurisdictions, such as in Sub-Saharan Africa. Third, understanding the interrelated nature of patent records within families can allow us to infer or "in fill" information about records from jurisdictions where only minimal data is reported. For example, if inventor data is omitted from one record but is included on two others, all of the same patent family. Fourth, the "web-like" structure of patent families suggests certain query strategies for identifying inventions globally. It is only necessary, for example, to identify one family member in a primary query. Then, it is possible to identify all related records that make up that invention's patent family.

In addition, when using patent family metrics from publicly available data sources, it is important to interpret the available statistics with an understanding of the types of families that are possible given the several potential combinations of invention origin and filing patterns. These possibilities are illustrated in Table 3. From the point of view of a given country or patent jurisdiction (called "country X" in Table 3) it is possible to consider patent families for inventions that originated in the country and separately to consider patent families for inventions that are filed in that office.

Table A1. The eight possible types of patent families in terms of invention origin and filing status from the point of view of a given country's patent office, designated here as country "X". Various combinations of these eight different types of patent families are reported in statistics from WIPO, regional, and national patent offices. Shaded boxes correspond to 'foreign oriented' patent families.

			INVENTION STATUS	
			Invention originated in X.<br>*(domestic or resident invention)*	***Invention originated outside X.***<br>*(foreign invention)*
FILING STATUS	***Filed in X***	***First filed in X***	**A. First filed in X.**<br>**Never filed outside X.**<br>(domestic-only patent families)	**E. First filed in X.**<br>**Never filed outside X.**
			**B. First filed in X.**<br>**Then also filed outside X.**	**F. First filed in X.**<br>**Then also filed outside X.**
		First filed outside X	**C. First filed outside X.**<br>**Then also filed in X.**	**G. First filed outside X.**<br>**Then also filed in X.**
	Never filed in X		**D. First filed outside X.**<br>**Never filed in X.**	H. Rest of world's inventions,<br>never filed in X

By Filing: Total patent families *filed* in X = A+B+C+E+F+G
"Foreign oriented" patent families *filed* in X = B+C+E+F+G
Total patent families *first filed* in X = A+B+E+F
"Foreign oriented" patent families *first filed* in X = B+E+F

By Origin: Total patent families for inventions *originated* in X = A+B+C+D
Domestic patent families for inventions *originated* in X = A
"Foreign oriented" patent families for inventions *originated* in X = B+C+D

The simplest differentiation often made in the literature is between "domestic" patent families and "international" or "foreign oriented" patent families. WIPO distinguishes between "domestic-only" and "foreign-oriented" patent families, based upon the country of origin of the invention. When the patent family consists only of filings made in the same country as the invention's origin (as determined by the address of the first listed applicant), it is considered a domestic patent family (box A in Table A1). If any of the documents in the patent family are filed in a country different from the invention's country of origin, the patent family is considered "foreign oriented" (shaded boxes, B, C, D, E, F, and G, in Table A1). More simplistically, at InSTePP we differentiate between patent families made up of documents from only one patent office (boxes A and E in Table A1), defined as "domestic" patent families, and patent families with documents from more than one patent office (boxes B, C, F, and G, for filings, in Table A1), defining these as "international patent families".

3. DATA ANALYSES

3.1 Comparing foreign-oriented patent families and the GDP of the country (or countries) served by a given patent office (Figure 2 in main text)

Data on GDP is taken from the World Bank's World Development Indicators. The specific measure used in this analysis is GDP at market prices (current US$). As with patent family counts above, average was taken of each country's count for the five years 2004-2008. This smooths out some of the volatility that occurs, especially for smaller and low-income countries, in this indicator.

Data on how many foreign oriented patent families originate from each country are from WIPO Statistical Database, searching Intellectual property right = Patent, and within the patent data selecting Indicator 7 "Foreign-oriented patent family by origin and destination office" and reporting "Total count by applicant's origin (equivalent count)". The average was taken of the count for the five years 2004-2008, again to smooth out some of the volatility that occurs for smaller and low-income countries.

Data on how many foreign oriented patent families are filed at each patent office are from WIPO Statistical Database, searching Intellectual property right = Patent, and within the patent data selecting Indicator 7 "Foreign-oriented patent family by origin and destination office" and reporting "Total count by filing office". The average was taken of each office's count for the five years 2004-2008.

For figure 2.a, values were matched by country between "average GDP" and "average count of patent families by applicant's origin" fort the five year and matches were retained for all countries in which complete data was available for both measures. Then, values were summed together for all member countries of the five regional patent offices that were active with that regional patent office between 2004 and 2008 (for example, members of that joined the European Patent Office after 2008 were not included in the calculation of GDP or patent families for the EPO. Finally, natural logs were taken of both sets of measures, due to the large dispersion of absolute values across countries. This allowed for a more compact plotting in the figure.

For figure 2.b, in a similar manner, values were matched by country between “average GDP” and “average count of patent families count by filing office” and retained for all countries in which data was available for both. Then, values were summed together for all member countries of the five regional patent offices. Natural logs were taken of both sets of measures.

3.2 Counting inventions filed in sub-Saharan Africa’s three main patent offices (Figure 3 in main text)

Data on total inventions filed in South Africa, OAPI, and ARIPO are from WIPO Statistical Database. No single indicator from WIPO, however, provides the total domestic and foreign patent families filed at each office. In order to compile this value, several manipulations were necessary.

We obtained Indicator 6 “Patent family by origin and first filing office” reporting “Total count by first filing office” for OAPI, ARIPO, and South Africa. According to our typology in Table A1 above, this encompasses patent families of types A, B, E, and F, all of the patent families first filed in each respective office, regardless of where they originated or whether they were also subsequently filed abroad (and thus came to be considered “foreign oriented” patent families). Thus, total patent families first filed at a given office = A+B+E+F.

We then obtained Indicator 7 “Foreign-oriented patent family by origin and destination office” reporting “Total count by (destination) filing office” for OAPI, ARIPO, and South Africa. According to our typology in Table A1 above, this encompasses patent families of types B, C, E, F, and G, all of the foreign-oriented patent families to be filed in each respective office, regardless of origin or of first-filing office. Thus, total foreign oriented patent families filed at a given office = B+C+E+F+G.

We also obtained Indicator 8 “Foreign-oriented patent family by origin and first filing office” reporting “Total count by filing office” for OAPI, ARIPO, and South Africa, which according to our typology in Table A1 above encompasses patent families of types B, E, and F. This reported figure sums all patents that were first filed at each of those three offices, whether they originated domestically (type B) or

originated from a foreign (or non-member) country (types E and F). Thus, foreign-oriented patent families first filed at a given filing office = B+E+F.

The number of domestic only patent families by year were calculated by taking the difference between total patent families by applicant's origin (A+B+E+F) and foreign oriented patent families by applicant's origin (B+E+F). Thus, domestic only patent families filed at a given office = A.

Finally, domestic only patent families (A) and total foreign oriented patent families (B+C+E+F+G) were combined for total patent families filed at a given office = A+B+C+E+F+G. These are the annual values of new patent families plotted for South Africa, OAPI, and ARIPO in Figure 3.a.

Data on biological inventions filed in each of the three offices was derived from the InSTePP Global Genetics Database first identifying all of the patent families in the database that included a document with a publication ID number that began with ZA, AP, or OA. Then, year of application for each of those documents was extracted, for those patent families that had more than one document from a given office, then the minimum year was extracted. Finally, subtotals by year obtained the annual values of new patent families plotted for South Africa, OAPI, and ARIPO in Figure 3.b.

3.3 Determining technologies being protected with patents in sub-Saharan Africa

Data on technology categories were obtained from the WIPO Statistics Database querying Indicator 4 "Patent publications by technology" and reporting "Total count by filing office" for South Africa, OAPI, and ARIPO. Publications by technology were then summed by category over the available years, sorted, and then top ten categories were reported, in the rank order of overall totals, for each year.

Figure A2. Annual shares of the ten most prevalent technology categories among patent publications at the three main sub-Saharan patent offices (a) South Africa, (b) ARIPO, and (c) OAPI.

Panel a. South Africa

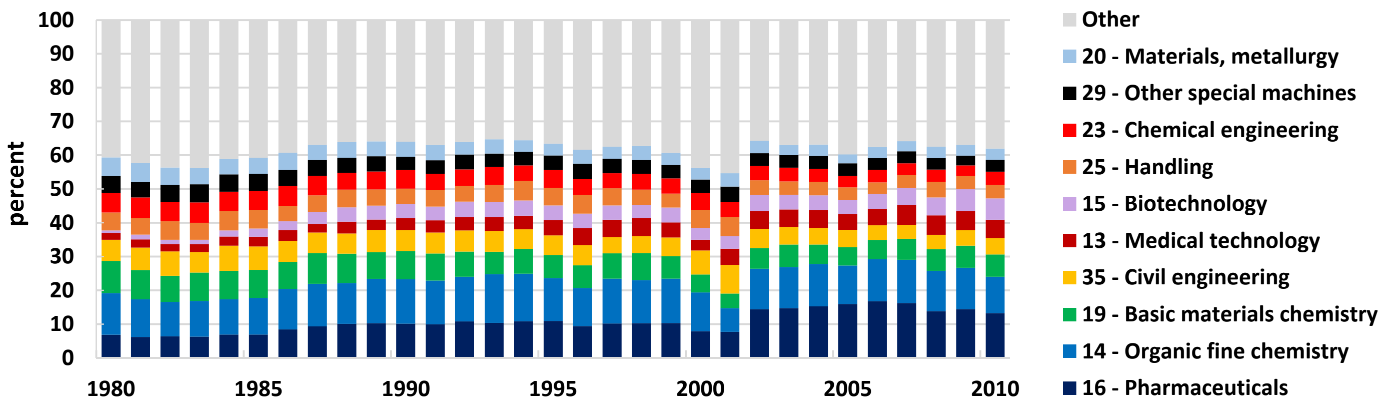

Panel b. ARIPO

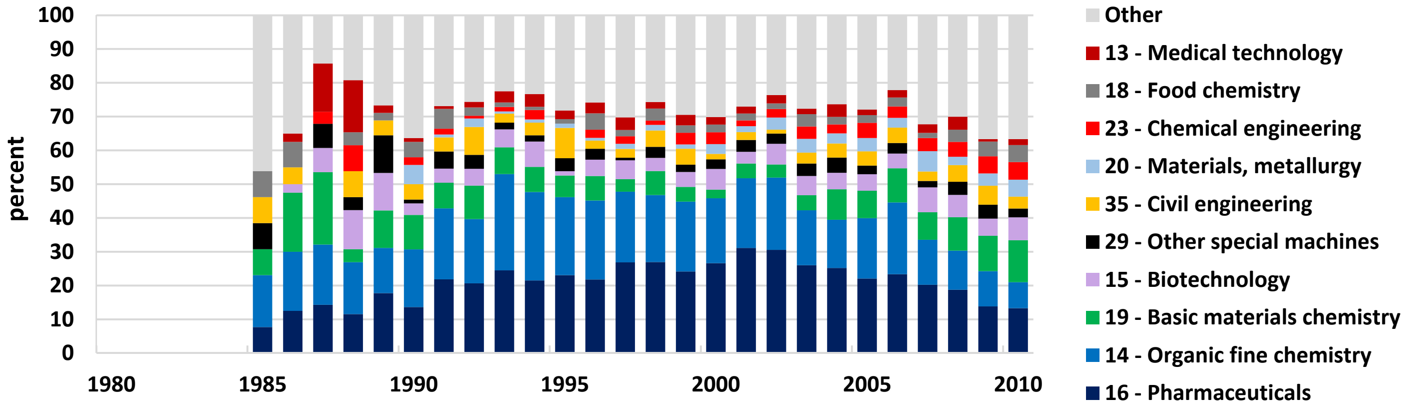

Panel c. OAPI

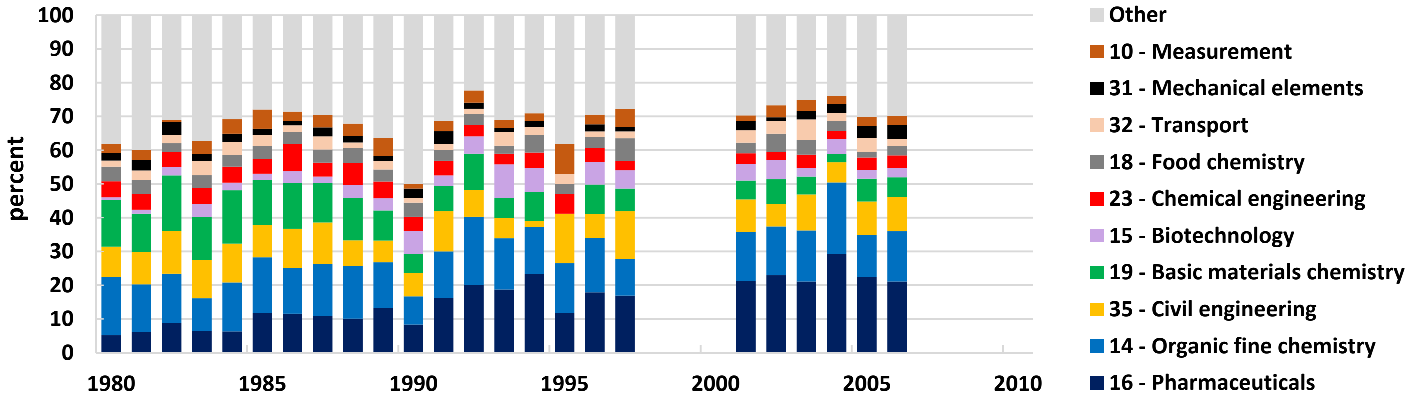

Data Source: WIPO Statistics Database, 2016

3.4 *Determining industry of application for biological inventions (Table 1 in main text)*

The InSTePP Global Genetics Patent Database utilizes Thomson Innovation's proprietary DWPI Manual Code classifications to determine high-level industries of application. Close to 10,000 DWPI Manual Code numbers were assigned by the authors at InSTePP to one or more industries, roughly following the following scheme:

- Section A = Chemistry
- Section B = Pharmaceuticals
- Section C = Agriculture
- Sections D01-D03, D05-06, D10 = Food & Beverage
- Section D04 plus selections B12-K04 (Environmental testing), D05-A04 (waste fermentation), and E11-Q (waste recovery, purification, treatment) = Environment
- Section F = Pulp & Paper
- Section H, plus selections from sections D04-D05, D10, E10-E11, L03, and X16 = Energy
- Sections from A12-V, B04-P, B11-C, C04, C12-K, C14, D03-G, D05-H16 (transgenic animals) = Veterinary

For this analysis a simple tally was run, counting the number of patent filings (54,194 filed in African patent offices or by African inventors) that was tagged in each of the high-level industry categories listed above. Since each invention can be tagged with multiple DWPI manual codes, this tally resulted in significant overlap between the high-level industry categories. Table A2 shows the shares of filings that represent various combinations of categories based on the DWPI Manual Code scheme. Significant degrees of overlap are observed between Pharma, Agriculture, and Veterinary and between Pharma and Industrial Chemistry.

Table A2. Cross table of shares of filings by industry of application, based on DWPI manual codes, for the 54,194 patent filings made in African patent offices

	Pharma-ceuticals	Agri-culture	Food & Beverage	Vet-erinary	Paper & Textile	Energy	Environ-ment	Industrial Chemistry
Pharma-ceuticals	***81.6%***							
Agriculture	13.1%	***17.7%***						
Food & Beverage	3.9%	1.7%	***6.0%***					
Veterinary	20.6%	10.1%	1.9%	***22.0%***				
Paper & Textile	2.7%	0.8%	0.4%	1.3%	***4.3%***			
Energy	3.1%	1.1%	1.3%	1.3%	0.6%	***6.2%***		
Environ-ment	2.7%	0.9%	0.5%	1.3%	0.5%	1.5%	***4.1%***	
Industrial Chemistry	20.1%	4.9%	3.2%	9.9%	3.1%	5.1%	2.8%	***31.8%***

3.5 Determining country of origin of patent families filed in sub-Saharan African offices (Figures 4.a, 4.b, and 4.c in main text)

Data on origins of patent filings received in the three main sub-Saharan African offices were drawn from WIPO Statistics Database, Indicator 7 “Foreign-oriented patent family by origin and destination office” reporting “Count by filing office and applicant's origin” selecting for the three major sub-Saharan African filing offices, from all countries of origin globally.

These were then augmented with annual counts of domestic only patent families which had been calculated by taking the difference between total patent families by applicant’s origin (A+B+E+F) and foreign oriented patent families by applicant’s origin (B+E+F), as explained in section II.B and Table A1, above.

Countries of origin were then summed and categorized according the following schema for the stacked-area timeline figure, in order to maintain domestic filings at the base, with filings originating from other

developing countries layered next, and then developed countries thereafter. The order is, from bottom to top:

- Developing
    - Domestic (member countries) filings
    - other Sub-Saharan Africa
    - other BRICs
    - all other developing
- Developed:
    - United States
    - Canada
    - France
    - United Kingdom
    - Germany
    - other European
    - Japan
    - Australia
    - other (non-European) OECD countries
    - unknown

3.6 Determining types of applicants/assignees

Data identifying the applicant/assignees is only available at the record level, and thus the analysis in this section relies on the InSTePP Global Genetics Database, which tags each applicant/assignee reported in the raw patent record data from Thomson Innovation according to whether it is a private sector entity (company or individual) or a public-sector entity (a government agency or publicly-funded, non-profit, or academic research institution), utilizing a set of linguistic algorithms. The InSTePP team also cleaned

names for the top 1000 applicant/assignees. These category dummy variables and cleaned applicant names were then tallied over the 54,194 patent families that constitute the sub-Saharan African partition of the data set.

Figure A3. Types of applicants/assignees on patent families in biological subject matters filed in Sub-Saharan Africa

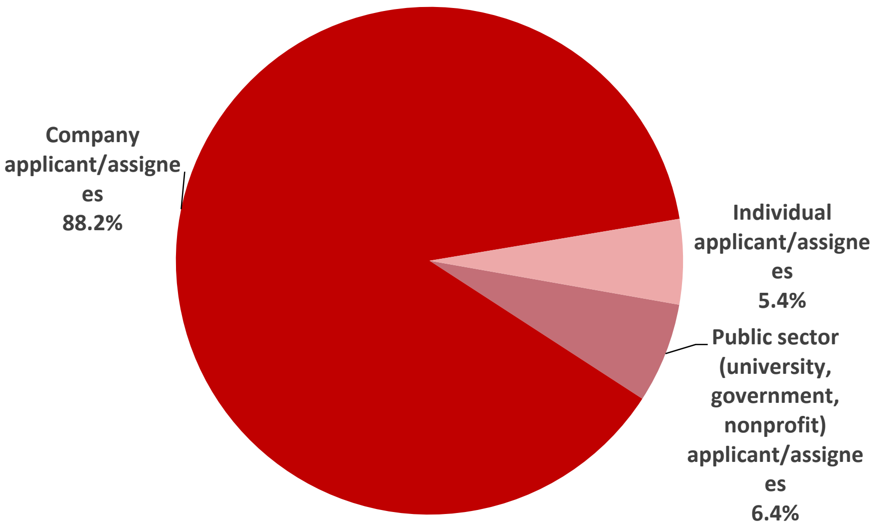

Data Source: InSTePP Global Genetics Database, 2016.

3.7 Determining inventions made by residents of sub-Saharan Africa (Figure 5 and Table 3 in the main text)

Data on the filing destinations of patents that originated in sub-Saharan Africa were drawn from WIPO Statistics Database, Indicator 7 "Foreign-oriented patent family by origin and destination office" reporting "Count by filing office and applicant's origin" selecting for inventions where the applicant's origin was a country of sub-Saharan Africa. Additional data were drawn from the InSTePP Global Genetics database of biological inventions, identifying inventions by country of inventor.

For Figure 5, panel a, counts by year were taken for total inventions where the applicant's origin was South Africa and counts were summed by year for total inventions where the applicant's origin was in any other country in the rest of sub-Saharan Africa.

For Figure 5, panel b, counts were tallied for South Africa and for all other countries (33 in total) of sub-Saharan Africa by year of first filing from the InSTePP Global Genetics database of biological inventions.

For Table 3, totals were taken by country across the entire available timeframe for each of three measures, due to the fact that for most countries data is missing or very small numbers prevail by year:

- Total count of inventions by country of ***applicant's origin***, from WIPO Statistics Database, Indicator 7 "Foreign-oriented patent family by origin and destination office" over the entire available time period from 1980 to 2010
- Total count of biological inventions, by country of ***inventor*** (based on registered inventors address data) from the InSTePP Global Genetics database of biological inventions over the entire available time period from 1970 to 2010
- Total count of biological inventions, by country of ***applicant or assignee***, from the InSTePP Global Genetics database of biological inventions over the entire available time period from 1970 to 2010

The largest number of inventions by applicants from a single country is observed for South Africa. Further analysis was undertaken for year-by-year analysis of inventions from South African applicants (see Figure A4) indicating a stable trend of about 1,000 to 1,200 inventions per year since 1980, with a decline in the counts after 2005. It cannot be determined whether the decline following 2005 is real or an artefact of data reporting lags (truncation).

Filing patterns of patent families for South African inventions remained remarkably stable over the three decades observed (Figure A4). Annually, about 80-90 percent of inventions that originated in South Africa were filed for patent protection in South Africa only. Another 10-15 percent that originated in South Africa were first filed in South Africa but then subsequently also filed abroad. Annually, a small share, about 3-5 percent, of inventions that originated in South Africa were first filed abroad and then may or may not have been subsequently filed in South Africa.

Figure A4. Filing patterns of inventions by residents of South Africa

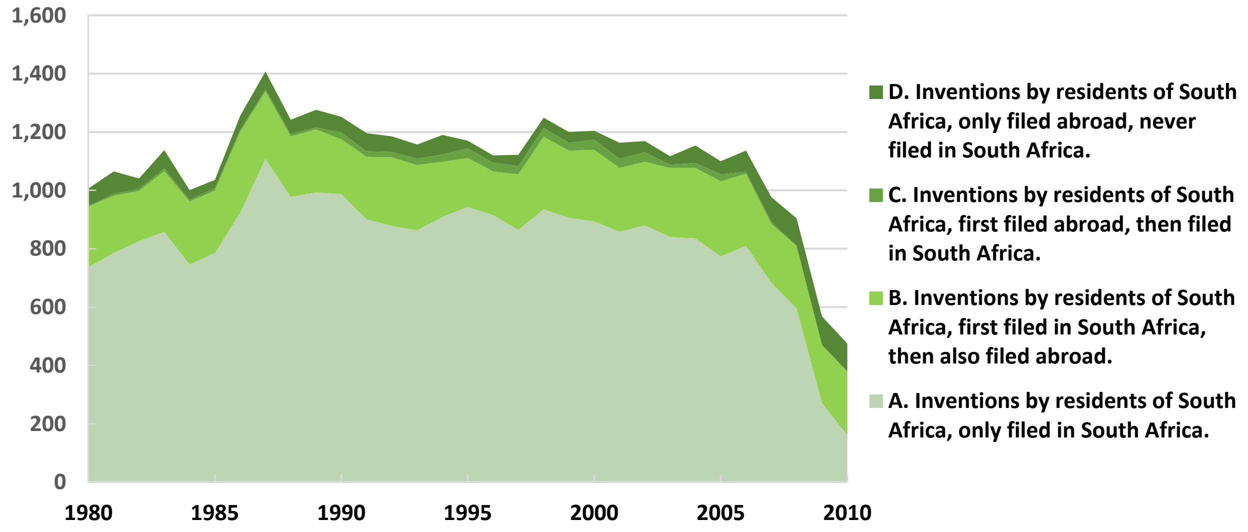

Notes: letters A, B, C, and D correspond to the different types of patent families represented in Table A1. It is not clear whether downward trends in years after 2006 (largely in type A inventions, those by residents of South Africa filed only in South Africa) are due to data truncation resulting from data reporting issues or are due to a secular decline in domestic filings by South African inventors.

Data Source: WIPO Statistics Database, 2016.

Figure A5. Annual numbers of new foreign-oriented patent family filings in patent offices worldwide on inventions made by residents of sub-Saharan Africa

Panel a. Numbers of patent family filings from inventions made in South Africa

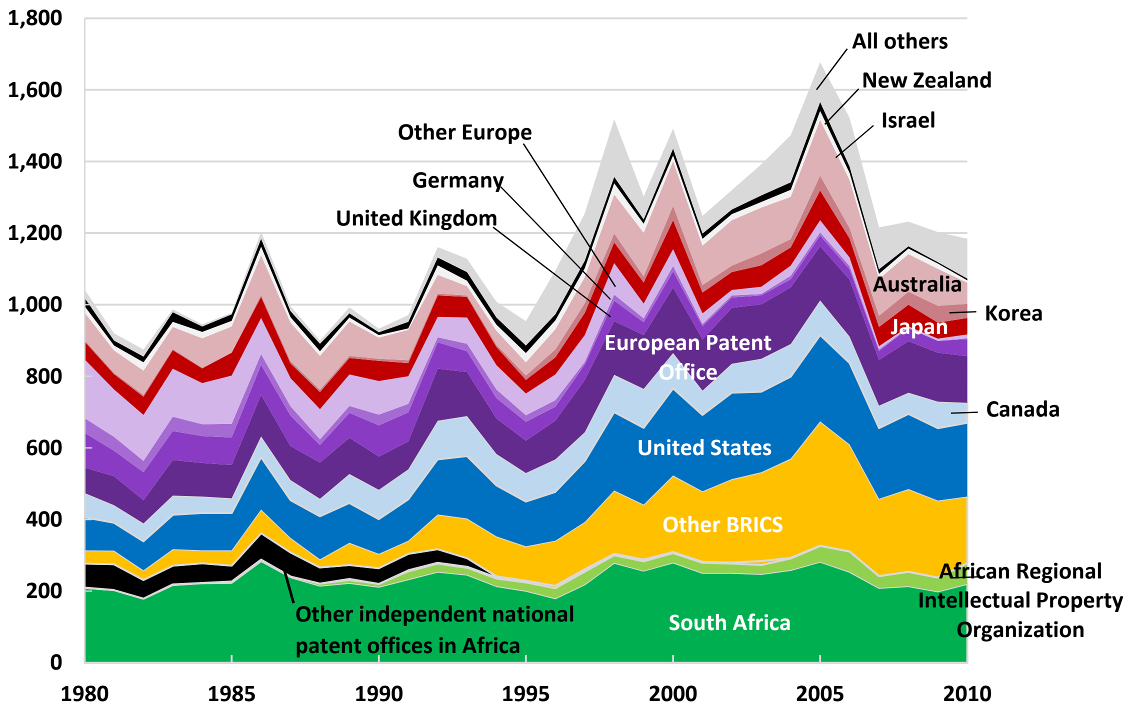

Panel b. Numbers of patent family filings from inventions made in all other countries of sub-Saharan Africa

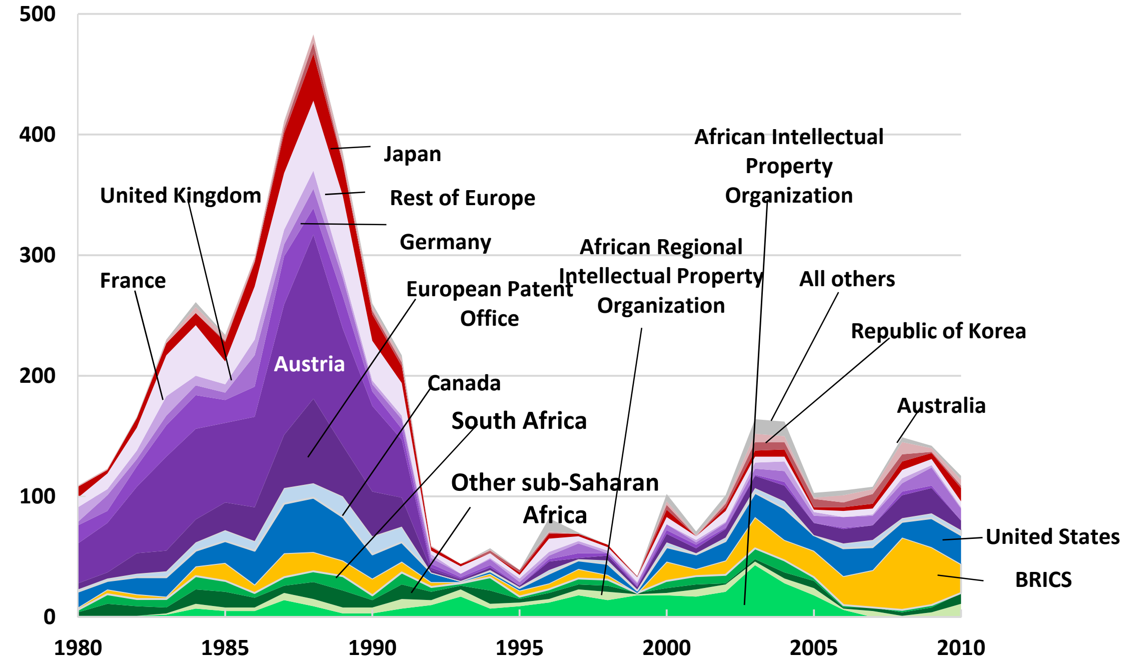

Data Source: WIPO Statistics Database, 2016.

Foreign filings on inventions that originated in South Africa (Figure A5, Panel a) and that originated in the rest of sub-Saharan Africa (Figure 5A, Panel b) were counted annually by country of destination and then categorized for the stacked-area timelines in Figure A5, which maintain domestic filings at the base, with filings originating from other developing countries layered next, and then developed countries thereafter.

Finally, we plotted out annual counts of biological inventions, by country of applicant or assignee for South Africa and then combined for all other sub-Saharan Africa, from the InSTePP Global Genetics database of biological inventions over the entire available time period from 1970 to 2010 (Figure A6). This analysis illustrates that, from a very small base, the numbers of African assignees have been growing steadily since about 1990 through the end of the time period.

Figure A6. Sub-Saharan African assignees, from South Africa and from all other Sub-Saharan African countries combined

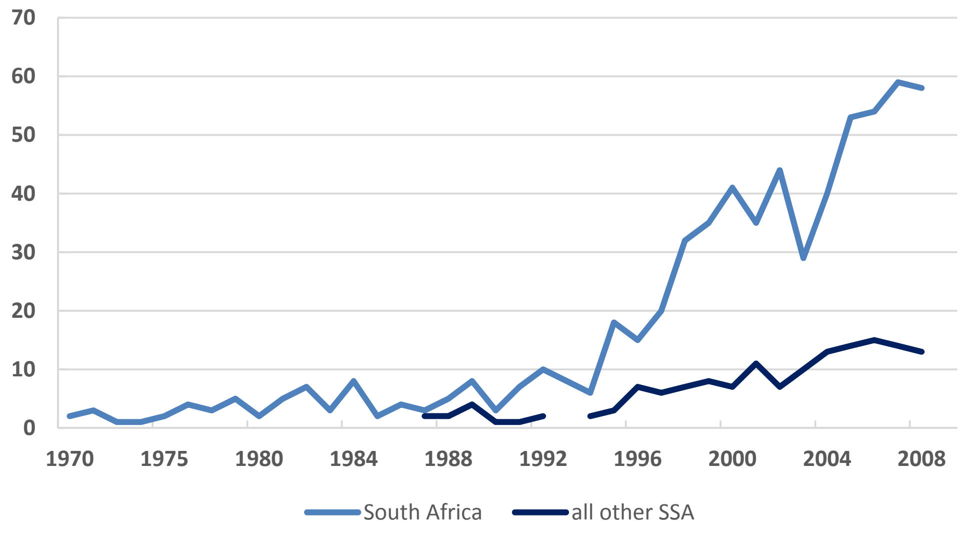

Data Source: InSTePP Global Genetics Database, 2016.
